# A Survey of Convolutional Neural Network in Breast Cancer

**DOI:** 10.32604/cmes.2023.025484

**Published:** 2023-03-09

**Authors:** Ziquan Zhu, Shui-Hua Wang, Yu-Dong Zhang

**Affiliations:** School of Computing and Mathematical Sciences, University of Leicester, Leicester, LE1 7RH, UK

**Keywords:** Breast cancer, convolutional neural network, deep learning, review, image modalities

## Abstract

**Problems:**

For people all over the world, cancer is one of the most feared diseases. Cancer is one of the major obstacles to improving life expectancy in countries around the world and one of the biggest causes of death before the age of 70 in 112 countries. Among all kinds of cancers, breast cancer is the most common cancer for women. The data showed that female breast cancer had become one of the most common cancers.

**Aims:**

A large number of clinical trials have proved that if breast cancer is diagnosed at an early stage, it could give patients more treatment options and improve the treatment effect and survival ability. Based on this situation, there are many diagnostic methods for breast cancer, such as computer-aided diagnosis (CAD).

**Methods:**

We complete a comprehensive review of the diagnosis of breast cancer based on the convolutional neural network (CNN) after reviewing a sea of recent papers. Firstly, we introduce several different imaging modalities. The structure of CNN is given in the second part. After that, we introduce some public breast cancer data sets. Then, we divide the diagnosis of breast cancer into three different tasks: 1. classification; 2. detection; 3. segmentation.

**Conclusion:**

Although this diagnosis with CNN has achieved great success, there are still some limitations. (i) There are too few good data sets. A good public breast cancer dataset needs to involve many aspects, such as professional medical knowledge, privacy issues, financial issues, dataset size, and so on. (ii) When the data set is too large, the CNN-based model needs a sea of computation and time to complete the diagnosis. (iii) It is easy to cause overfitting when using small data sets.

## 1 Introduction

For people all over the world, cancer is one of the most feared diseases and one of the major obstacles to improving life expectancy in countries around the world [1–3]. According to the survey, cancer is one of the biggest causes of death before the age of 70 in 112 countries. At the same time, cancer is the third and fourth leading cause of death in 23 countries [4–7].

Among all kinds of cancers, breast cancer is the most common cancer for women [8–12]. According to the data from the United States in 2017, there were more than 250,000 new cases of breast cancer [13]. 12% of American women may get breast cancer in their lifetime [14]. The data surveyed in 2020 showed that female breast cancer had become one of the most common cancers [4].

A large number of clinical trials have proved that if breast cancer is diagnosed at an early stage, it will give patients more treatment options and improve the treatment effect and survival ability [8,15–17]. Therefore, there are many diagnostic methods for breast cancer, such as biopsy [18].

**Figure 1:**
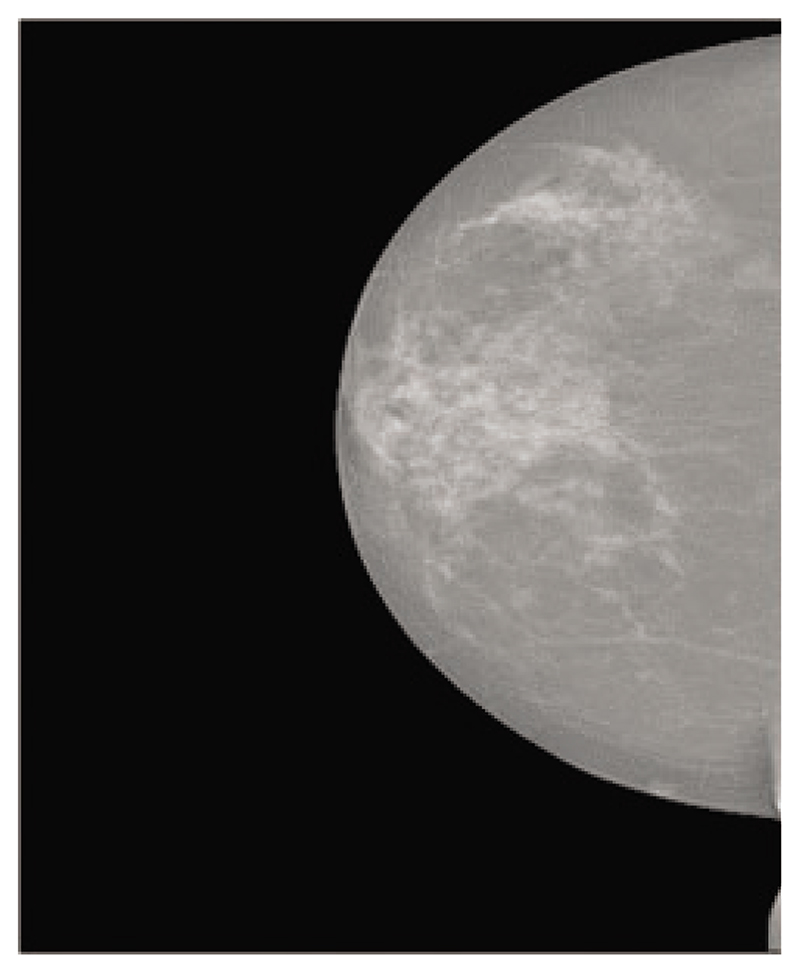
The breast cancer image [22]

The image of breast cancer is shown in Fig. 1. Invasive carcinoma and carcinoma *in situ* are two types of breast cancer [19]. Carcinoma *in situ* cannot be upgraded in the body. About one-third of new breast cancer is carcinoma *in situ* [20]. Most newly diagnosed breast cancer is invasive. Invasive cancer begins in the mammary duct and can spread to other breast sites [21].

**Figure 2:**
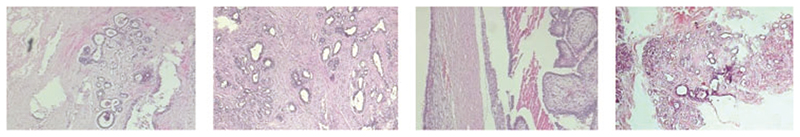
The images of the benign tumors

**Figure 3:**
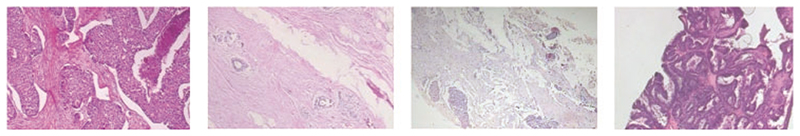
The images of the malignant tumors

Sometimes, the breast cancer image could be divided into two categories, which are benign and malignant. The images of benign tumors and malignant tumors are given in Figs. 2 and 3. Several imaging modalities are used for the diagnosis and analysis of breast cancer [23–25]. The abbreviated imaging modality table is given in Table 1. (i) Screen-film mammography (SFM) is one of the most important imaging modalities for early breast cancer detection [26]. But SFM also has some disadvantages. First, the sensitivity of SFM is low for the detection of the breast with dense glandular tissue [27]. This disadvantage may be caused by the film. Because once the film is finished, it is impossible to improve it. So sometimes there are pictures with low contrast [28]. Furthermore, SFM is not digital. (ii) Digital mammography (DM) is one of the effective imaging modalities for early breast cancer detection [29,30]. At the same time, DM has always been the standard imaging modality for female breast cancer diagnosis and detection [31]. However, DM has some limitations. The specificity of DM is low, which could cause some biopsies [32]. Another limitation of DM is that patients may face high radiation exposure [27]. This may cause some health hazards to patients. (iii) Magnetic resource imaging (MRI) is suitable for clinical diagnosis and high-risk patients [33]. MRI is very sensitive to breast cancer [20]. MRI still has some problems. Compared with DM, the MRI detection cost is higher [34]. Although MRI has high sensitivity, its specificity is low [35]. (iv) Ultrasound (US) is one of the most common methods for the detection of breast cancer. The US has no ionizing radiation [36]. Therefore, compared with SFM and DM, the US is safer and has lower costs [37]. But the US is an imaging modality that depends on the operator [38]. Therefore, the success of the US in detecting and differentiating breast cancer lesions is largely affected by the operator. (v) Digital breast tomosynthesis (DBT) is a different imaging modality. Compared with traditional mammography, DBT can take less time for imaging [39] and provide more details of the dense chest [40]. One problem with DBT is that DBT may not detect malignant calcification when it is at the slice plane [41]. It also takes more time to read than DM [42]. (vi) Histopathological images (HP) can capture information about cell shape and structural information [43]. However, it is invasive and requires additional costs [44]. The details of these different imaging modalities are presented in Table 2.

Medical imaging is usually done manually by experts (pathologists, radiologists, etc.) [45]. Through the above overview of several medical imaging, there are some problems in medical imaging [46]. Firstly, experts are required to manually analyze medical imaging, but there are few experts in this field in many developing countries [47]. Secondly, the process of manual analysis of medical imaging is very long and cumbersome [48]. Thirdly, when experts manually analyze medical imaging, they can be influenced by foreign factors, such as lack of rest, decreased attention, etc. [27].

With the continuous progress of computer science, computer-aided diagnosis (CAD) models for breast cancer have become a hot prospect [49]. Scientists have been studying CAD models for breast cancer for more than 30 years [50,51]. CAD models for breast cancer have the following advantages [52]: (i) CAD models can improve specificity and sensitivity [53]. (ii) Unnecessary examinations can be omitted by CAD models [54]. This can shorten the diagnosis time and reduce the cost. (iii) The CAD models can reduce the mortality rate by 30% to 70% [13]. With the development of computing power, the convolutional neural network (CNN) is one of the most popular methods for the diagnosis of breast cancer [55–57]. Recently, a sea of research papers has been published papers about breast cancer based on CNN [58–61]. However, these research papers only propose one or several methods, which cannot make readers fully understand the diagnosis technology of breast cancer based on the CNN model. Therefore, we complete a comprehensive review of the diagnosis of breast cancer based on CNN after reviewing a sea of recent papers. In this paper, readers can not only see the CNN-based diagnostic methods for breast cancer in recent decades but also know the advantages and disadvantages of these methods and future research directions. The main contributions of this survey are given as follows:

- A sea of major papers about the diagnosis of breast cancer based on CNN is reviewed in this paper to provide a comprehensive survey.
- This survey presents the advantages and disadvantages of these state-of-the-art methods.
- A presentation of significant findings gives readers the opportunities available for future research.
- We give the future research direction and critical challenges about the CNN-based diagnostic methods for breast cancer.

The rest structure of this paper is shown as Section 2 talks about CNN. Section 3 introduces the breast cancer data set. Section 4 presents the application of CNN in breast cancer. The conclusion is given in Section 5.

## 2 Convolutional Neural Network

**Figure 4:**
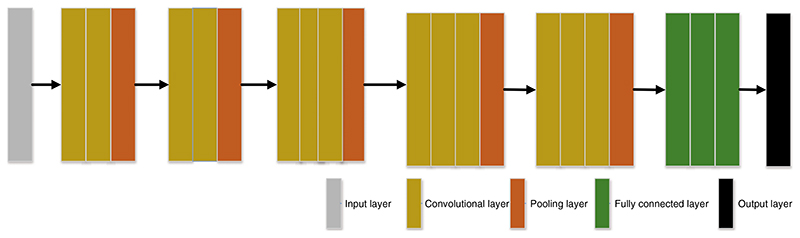
The architecture of VGG16

**Figure 5:**
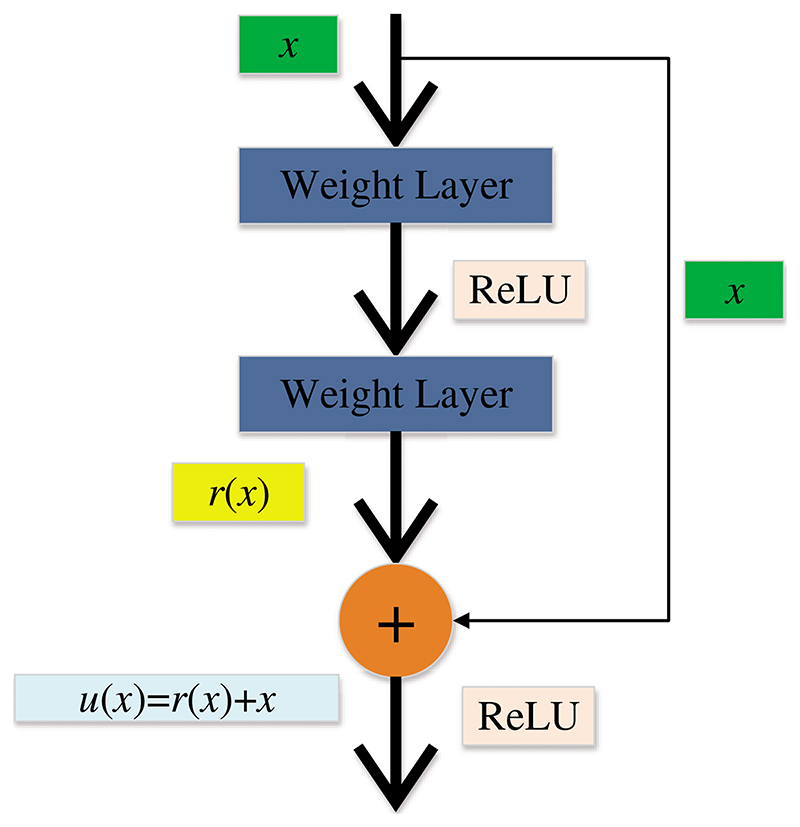
The residual learning

**Figure 6:**
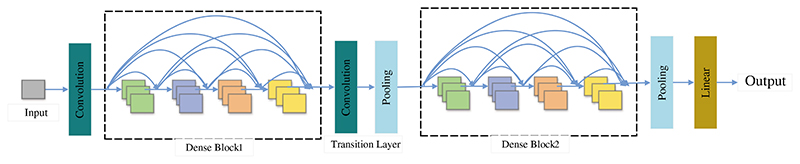
The DenseNet block

In the past few decades, the importance of medical imaging has been fully verified [62–66]. With medical imaging, people can help detect, diagnose and treat early diseases [33,67–69]. However, as analyzed above, medical imaging still has some shortcomings [70–73]. With the progress of CNN technology, lots of researchers use CNN to diagnose breast cancer [74–77]. A large number of studies have proved that CNN shows superior performance in breast cancer diagnosis [78–81]. CNN can be a solution for the continuous improvement of image analysis technology and transfer learning [82–84]. Recently, a large number of researchers take CNN as the backbone model for transfer learning, such as ResNet, AlexNet, DenseNet, and so on [85–87]. Some layers of CNN models are frozen, and the unfrozen layers are retrained with the data set [88–90]. Sometimes researchers use CNN models as feature extractors and select other networks as the classifiers [91–93], such as support vector machines (SVM) [94], randomized neural networks (RNNs) [95], etc. At present, lots of CNN models are used in breast cancer diagnosis [96], such as AlexNet, VGG, ResNet, U-Net, etc. [93,97,98]. CNN is a computing model composed of a sea of layers [99–102]. Fig. 4 shows the structure of a classic CNN model-VGG16 [103]. The residual learning and DenseNet block are given in Figs. 5 and 6.

The convolution layer is one of the most important components of CNN and usually connects the input layer [104–108]. The input is scanned by the convolution layer based on the convolution kernel for extracting features. Different convolution kernels will extract different features in the same input layer [109]. There may be multiple convolution layers in a CNN model [110]. Basic features are usually extracted by the front convolution layers. The convolution layers in the back are more likely to extract advanced features [88].

We first define the parameters of the convolution layer: the input image size is *I* × *I*, the convolution kernel is *K* × *K*, *S* represents the stride, the padding is *P*, and the output size is *O*×*O*. Padding refers to additional pixels used to supplement the zero value around the input image [104,111–113]. Stride refers to the step size of each convolution kernel sliding [114–116]. The formula is shown below:

(1) $$O=\frac{I-K+2P}{S}+1$$

**Figure 7:**
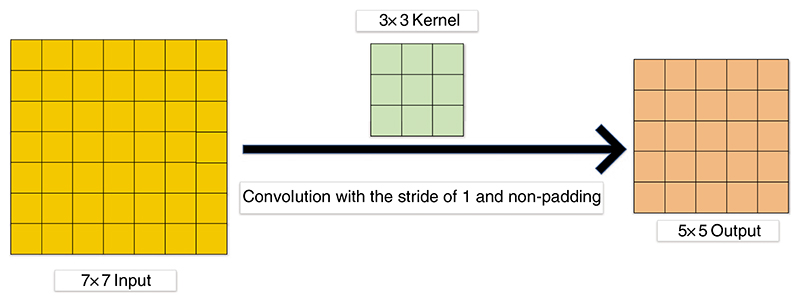
A sample of convolution

Fig. 7 gives a sample of convolution. In Fig. 7, the stride and padding are set as 1 and 0, respectively. *I*=7, *K*=3, *P*=0, *S*=1, thus *O*=5.

**Figure 8:**
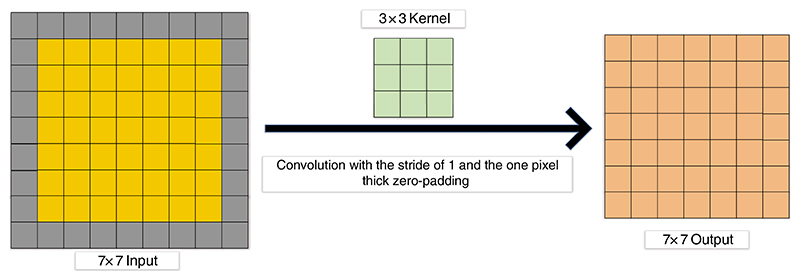
Convolution with the one-pixel thick zero-padding

More and more researchers use zero padding [117] in the convolution layer. In Fig. 8, the output size is the same as the input size with the one zero-padding.

The features from the input are extracted by the convolution layer [118–121]. After multiple convolutions, the feature dimension becomes higher and higher, resulting in too much data [122]. But too much data may contain too much redundant information [122–124]. This redundant information will not only increase the amount of training but also lead to overfitting problems [123,125–127]. At this time, some researchers could select the pooling layer to downsample the extracted features. The main functions of the pooling layer are (i) translation invariance and (ii) feature dimensionality reduction [124].

**Figure 9:**
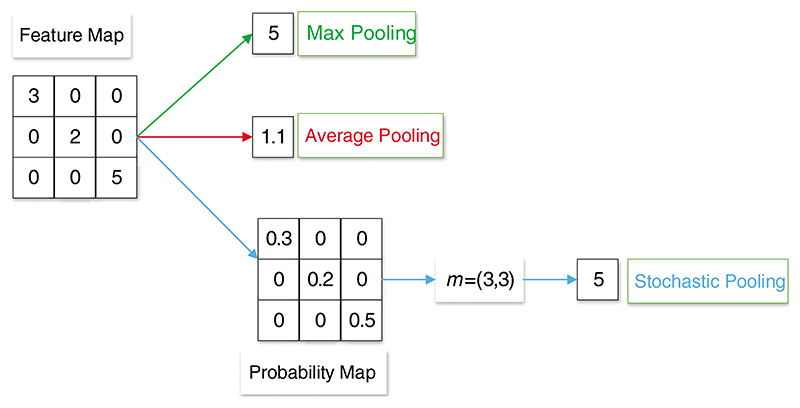
An example of max, average, and stochastic pooling

At present, the three main pooling methods are max pooling [128], average pooling [129], and stochastic pooling [130], as given in Fig. 9.

*A_R_* is pooling region *R* in the feature map and *k* is the index of each element within it. The function is set as *pool* ():

(2) $$p=pool(a_{k}),\;k\in A_{R}$$

Max pooling is to obtain the maximum value of pixels in the specific area of the feature map in a certain step [129]. The formula of max pooling (*p_m_*) is as follows:

(3) $$p_{m}=\text{max}(a_{k}),\;k\in A_{R}$$

Average pooling is to average the pixels in a specific area of the feature map in a certain step [131]. The formula of average pooling (*p_a_*)is as follows:

(4) $$p_{a}=\frac{\sum_{k\in A_{R}}a_{k}}{\left|A_{R}\right|}$$

where |*A_R_*| means the number of elements in *A_R_*.

Stochastic pooling selects the map response based on the probability map *B* = (*b*_1_, *b*_2_ … *b_k_*, …) [132]. The formula of *b_k_* is as follows:

(5) $$b_{k}=\frac{a_{k}}{\sum_{k\in A_{R}}a_{k}}$$

Stochastic pooling outputs are picked from the multinomial distribution. The formula of stochastic pooling (*p_s_*) is as follows:

(6) $$p_{s}=a_{m},\;\text{where}\;m\sim (b_{1},b_{2}\ldots b_{k},\ldots )$$

The nonlinearity is introduced into CNN through activation. Two traditional activation functions are Sigmoid [133] and Tanh [134]. The equation of Sigmoid is given as:

(7) $$\text{Sigmoid}\;(x)=\frac{1}{1+e^{-x}}$$

The Tanh is written as:

(8) $$\text{Tanh}\;(x)=\frac{e^{x}-e^{-x}}{e^{x}+e^{-x}}$$

These two traditional activation functions do not perform well in convergence. The rectified linear unit (ReLU) [135] accelerates the convergence. The equation of ReLU is as follows:

(9) $$\text{ReLU}(x)=\{\begin{array}{l} x,x>0\\ 0,x\leq 0 \end{array}$$

There are some problems with the ReLU. When *x* is less than or equal to 0, the activation value is 0. In this case, leaky ReLU (LReLU) [136] is proposed. Compared with ReLU, when *x* is less than or equal to 0, the activation value is a small negative. The equation of LReLU is given as:

(10) $$\text{LReLU}(x)=\{\begin{array}{l} x,x>0\\ 0.01x,x\leq 0 \end{array}$$

Based on LReLU, researchers proposed PReLU [137]. When *x* is less than or equal to 0, the slope is learned adaptively from the data. The PReLU is shown as:

(11) $$\text{PReLU}(x)=\{\begin{array}{l} x,x>0\\ zx,x\leq 0 \end{array}$$

where *z* is very small and decided by other parameters.

Each activation function has its characteristics, which is shown in Table 3.

The CNN model maps the input data to the feature space with the convolution layer, pooling layer, and activation function. The function of the fully connected layer is to map these to the sample space. The fully connected layer convolutes the feature map to obtain a one-dimensional vector, weighs the features, and reduces the spatial dimension.

CNN may consist of multi-layer fully connected layers. Global average pooling is proposed to substitute the fully connected layer, which greatly reduces parameters. However, global average pooling does not always perform better than the fully connected layer, such as in transfer learning.

The increasing depth of the CNN model increases the difficulty of adjusting the model. The input of each subsequent layer changes in the training. In this case, this could cause the disappearance of the gradient of the low-level network. The reason why the neural structure of a deep neural network converges more and more slowly is the gradient disappearance [138].

Batch normalization adjusts the input value of each layer to the standard normal distribution. The data is set as:

(12) $$X=[x_{1},x_{2},\ldots ,x_{n}]$$

Firstly, calculate the mean value of batch *B*:

(13) $$\varphi_{B}=\frac{1}{n}\sum_{i=1}^{h}x_{i}$$

Secondly, calculate the variance:

(14) $$\vartheta_{B}=\frac{1}{n}\sum_{i=1}^{h}{\left(x_{i}-\varphi_{B}\right)}^{2}$$

Thirdly, perform the normalization:

(15) $$x_{i}^{\prime }=\frac{x_{i}-\varphi_{B}}{\sqrt{\vartheta_{B}^{2}+\in }}$$

where ∈ is greater than 0, which makes sure that the denominator is greater than 0.

Finally, two parameters are proposed to increase network nonlinearity:

(16) $$y_{i}=\alpha x_{i}^{\prime }+\beta$$

where *α* is the scale parameter and *β* is the shift parameter.

**Figure 10:**
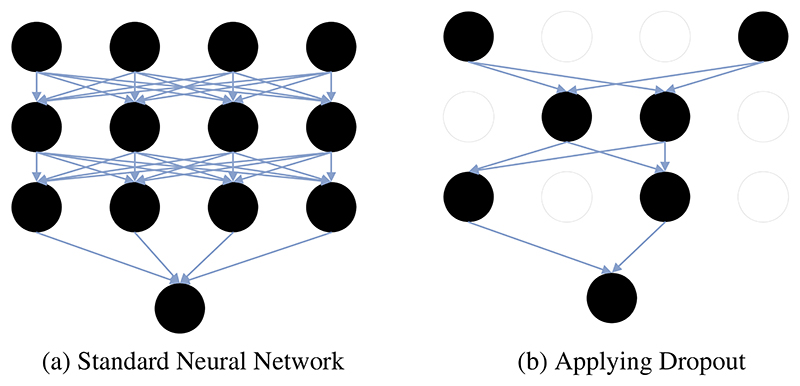
An example of the dropout

In the CNN model, too few training samples could lead to the overfitting problem. The overfitting problem is that the loss function of the CNN model is small and high accuracy is obtained during training, but the loss function is large, and the accuracy is low during testing. In this case, researchers usually select the dropout to prevent overfitting problems. In CNN model training, some nodes in the hidden layer are set as 0, as shown in Fig. 10. This reduces the interaction between hidden layers [139].

One of the important indexes used to evaluate the performance of a CNN model is the confusion matrix The confusion matrix is given in Table 4.

TP, FN, FP, and TN are true positive, false negative, false positive, and true negative, respectively.

However, the confusion matrix only counts numbers. Sometimes in the face of lots of data, it is difficult to measure the quality of the model simply by counting the numbers. Therefore, there are several other indicators for the basic statistical results.

1. Accuracy: It means the proportion of all samples with correct prediction.
(17) $$\text{Accuracy}=\frac{\text{TP}+\text{TN}}{\text{TP}+\text{FP}+\text{TN}+\text{FN}}$$
2. Sensitivity (TPR): It indicates the proportion of positive cases recognized as positive cases in the positive cases
  (18) $$\text{Sensitivity}=\frac{\text{TP}}{\text{TP}+\text{FN}}$$
3. Specificity: It represents the proportion of negative cases recognized as negative cases in the negative cases.
(19) $$\text{Specificity}=\frac{\text{TN}}{\text{FP}+\text{TN}}$$
4. Precision: It Indicates how many samples with positive predictions are positive.
(20) $$\text{Precision}=\frac{\text{TP}}{\text{TP}+\text{FP}}$$
5. F1-measure: It is the harmonic average of precision and recall.
(21) $$\text{F1}=\frac{\text{2TP}}{\text{2TP}+\text{FP+FN}}$$
6. FPR: When the result is negative, it predicts a positive value.
(22) $$\text{FPR}=\frac{\text{FP}}{\text{TN+FP}}$$
7. Receiver Operating Characteristic (ROC) curve: TPR and FPR are the y-axis and x-axis, respectively. From the definitions of FPR and TPR, it can be understood that the higher the TPR and the smaller the FPR, the more efficient the CNN model will be.
8. Area under Curve (AUC): It is between 0 and 1 and means the area under ROC. The model would be better with the larger AUC.
9. The Dice Similarity Coefficient (DSC) is usually used as the measurement to evaluate the quality of the segmentation. The DCS measures the overlap between manual segmentation (*M*) and automated segmentation (*A*).
  (23) $$\text{DSC(}A,M\text{)}=\frac{2\left|A\cap M\right|}{\left|A\right|+\left|M\right|}$$
  where |*A* ⋂ *M*| represents the intersection of *A* and *M*.
10. The Mean Absolute Error (MAE) is the average distance between the predicted (*t*) and the truth (*y*) of the sample.
  (24) $$\text{MAE}=\frac{1}{m}\sum_{i=1}^{m}\left|t_{i}-y_{i}\right|$$
  where *m* is the number of samples.
11. The Intersection over Union (IoU) evaluates the distance between the predicted value (*V*) and the ground truth (*G*).
  (25) $$\text{IoU}=\frac{\left|V\cap G\right|}{\left|V\cup G\right|}$$
  where |*V* ∪ *G*| means the area of union.

## 3 Common Datasets

In recent years, a lot of data sets were produced and published. Researchers can use some of them for research. Table 5 shows the details of some public data sets.

For DDSM, all images are 299 × 299. The DDSM project is a collaborative effort at the Massachusetts General Hospital (D. Kopans, R. Moore), the University of South Florida (K. Bowyer), and Sandia National Laboratories (P. Kegelmeyer). Additional cases from Washington University School of Medicine were provided by Peter E. Shile, MD, Assistant Professor of Radiology, and Internal Medicine. There are a total of 55890 samples in the DDSM dataset. 86% of these samples are negative, and the rest are positive. All data is stored as tfrecords files.

The images in the CBIS-DDSM (Curated Breast Imaging Subset of DDSM) are divided into three categories: normal, benign, and malignant cases. This data set contains a total of 4067 images. The CBIS-DDSM collection includes a subset of the DDSM data selected and curated by a trained mammographer. The images have been decompressed and converted to DICOM format.

The Mammographic Image Analysis Society (MIAS) Database contains 322 images. Each image in this dataset is 1024 × 1024. MIAS is an organization of UK research groups interested in the understanding of mammograms and has generated a database of digital mammograms. Films taken from the UK National Breast Screening Programme have been digitized to a 50-micron pixel edge with a Joyce-Loebl scanning microdensitometer, a device linear in the optical density range 0–3.2, and representing each pixel with an 8-bit word.

The INbreast database contains 410 breast cancer images. The INbreast database is a mammographic database, with images acquired at a Breast Centre, located in Hospital de São João, Breast Centre, Porto, Portugal. These images were obtained from 115 patients. Among these 115 patients, 90 were women with double breasts, and the other 25 were mastectomies. Each double breast patient would have four images, and each mastectomy patient would have two images.

The Breast Cancer Histopathological Image Classification (BreakHis) consists of 5429 malignant samples and 2480 benign samples. So, there are 9109 samples in the BreakHis data set. This database has been built in collaboration with the P&D Laboratory–Pathological Anatomy and Cytopathology, Parana, Brazil These microscopic images of breast tumor tissue were collected from 82 patients using different magnifying factors (40×, 100×, 200×, and 400×).

## 4 Application of CNN in Breast Cancer

This diagnosis of breast cancer through CNN is generally divided into three different tasks: 1 Classification; 2 Detection; 3 Segmentation. Therefore, this section is presented in three parts based on three different tasks.

### 4.1 Breast Cancer Classification

In recent years, the CNN model has been proven to be successful and has been applied to the diagnosis of breast cancer [140]. Researchers would classify breast cancer into several categories based on CNN models. We would review the classification of breast cancer based on CNN in this section.

**Figure 11:**
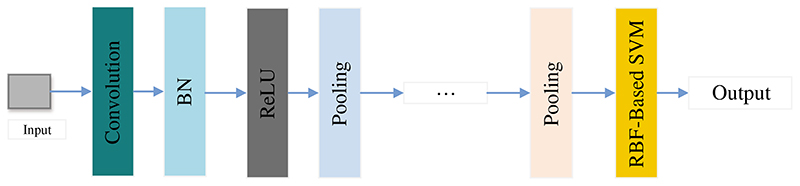
The structure of CNN+SVM

Alkhaleefah et al. [141] introduced a model combining CNN and support vector machine (SVM) classifier with radial basis function (RBF) for breast cancer image classification, as shown in Fig. 11. This method was roughly separated into three steps: Firstly, the CNN model was trained through breast cancer images. Secondly, the CNN model was fine-tuned based on the data set. Finally, the features extracted by the CNN model would be used as the input to RBF-Based SVM. They evaluated the proposed method based on the confusion matrix.

Liu et al. [142] introduced the fully connected layer first CNN (FCLF-CNN) method. This method added the fully connected layer before the convolution layer. They improved structured data transformation in two ways. The encoder in the first method was the fully connected layer. The second method was to use MSE losses. They tested different FCLF-CNN models and four FCLF-CNN models were ensembled. The FCLF-CNN model got 99.28% accuracy, 98.65% sensitivity, and 99.57% specificity for the WDBC data set, and 98.71% accuracy, 97.60% sensitivity, and 99.43% specificity for the WBCD data set.

Gour et al. [143] designed a network to classify breast cancer (ResHist). To obtain better classification results, they proposed a data enhancement technique. This data enhancement technique combined affine transformation, stain normalization, and image patch generation. Experiments show that ResHist had better classification results than traditional CNN models, such as GoogleNet, ResNet50, and so on. This method finally achieved 84.34% accuracy and 90.49% F1.

Wang et al. [144] introduced a hybrid CNN and SVM model to classify breast cancer. This method uses the VGG16 network as the backbone model. Because the data set was small, transfer learning was used in the VGG16 network. On the data set, they used the method of multi-model voting to strengthen the graph. At the same time, the image was also deformed. The accuracy of this method was 80.6%.

Yao et al. [145] introduced a new model to classify breast cancer. Extracting features from breast cancer images was based on CNN (DenseNet) and RNN (LSTM). Then the perceptron attention mechanism based on natural language processing (NLP) was selected to weight the extracted features. They used the targeted dropout in the model instead of the general dropout. They achieved 98.3% accuracy, 100% precision, 100% recall, 100% F1 for Bioimaging2015 Dataset.

Ibraheem et al. [24] proposed a three-parallel CNN branch network (3PCNNB-Net) to classify breast cancer. The 3PCNNB-Net was separated into three steps. The first step was mainly feature extraction. There were three parallel CNN to extract features. The three CNN models were the same. The second step was to use the average layer to merge the extracted features. The flattened layer, BN, and softmax layer were used as the classification layer. The 3PCNNB-Net achieved 97.04% accuracy, 97.14% sensitivity, and 95.23% specificity.

**Figure 12:**
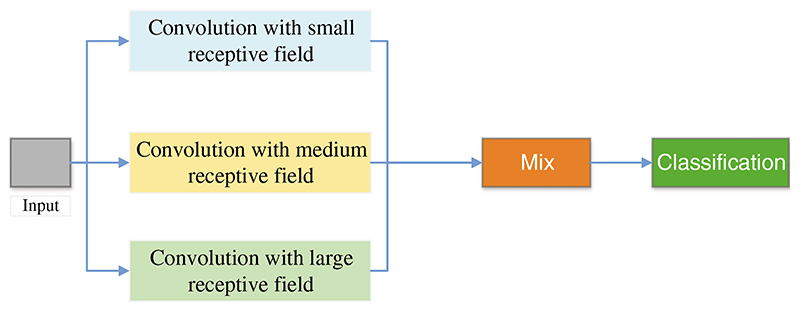
The structure of MA-CNN

Agnes et al. [146] proposed a multiscale convolutional neural network (MA-CNN) to classify breast cancer, as presented in Fig. 12. They used extended convolution and used three dilated convolutions of different sizes to extract different levels’ features. At this time, these features were combined.

Zhang et al. [115] designed an 8-layer CNN network for breast cancer classification (BDR-CNN-GCN). This network mainly consisted of three innovations. The first innovation was that they integrated BN and dropout. Second, they use rank-based stochastic pooling (RSP) instead of general maximum or average pooling. Finally, it was combined with two layers of graph convolutional network (GCN).

Wang et al. [147] introduced a breast cancer classification model according to CNN. In this paper, they selected inception-v3 as the backbone model for feature extraction of breast cancer images. And they did transfer learning to the inception-v3. This model got 0.886 sensitivity, 0.876 specificity, and 0.9468 AUC, respectively.

Saikia et al. [148] compared different classical CNN models in breast cancer classification. These classic CNN models used in this article were VGG16, VGG19, ResNet-50, and GoogLeNet-V3. The data set contained a total of 2120 breast cancer images.

Mewada et al. [149] introduced a new CNN-based model to classify breast cancer. In this new model, they added the multi-resolution wavelet transform. Spectral features were as important as spatial features in classification. Therefore, they integrated the features extracted from Haar wavelet with spatial features. They tested the new model on the BreakHist dataset and BCC2015 and obtained 97.58% and 97.45% accuracy, respectively.

**Figure 13:**
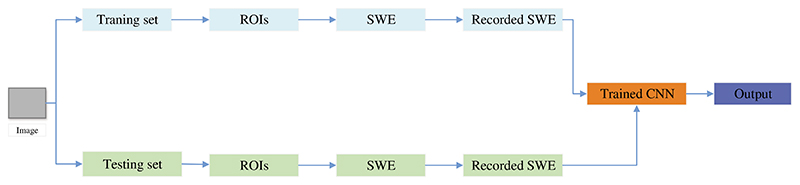
The structure SWE+CNN

Zhou et al. [150] proposed a new model for automatically classifying benign and malignant breast cancer, as shown in Fig. 13. This model can directly extract features from images, thus eliminating manual operation and image segmentation. This method combined shear wave elastography (SWE) and the CNN model to classify breast cancer. This SWE-CNN model produced 95.7% specificity, 96.2% sensitivity, and 95.8% accuracy, respectively.

Lotter et al. [151] introduced a multi-scale CNN for the classification of breast cancer. Firstly, the classifier was trained by segmenting the lesions in the image. Moreover, they trained the model by using the extracted features. They tested the multi-scale CNN on the DDSM dataset and obtained 0.92 AUROC.

Vidyarthi et al. [152] introduced a classification model combining CLAHE and CNN models for microscopic imaging of breast cancer. They tested the image preprocessing using CNN and without CNN. In this paper, they selected the BreakHist data set for testing. Finally, the hybrid model of CNN can get better classification results, which produces an accuracy of about 90%.

Hijab et al. [153] used a classical CNN model (VGG16) for breast cancer classification. They did some modifications to the VGG16. First, they selected the pre-trained VGG16 as the backbone model. Then they fine-tuned the backbone model. When fine-tuning, they froze all convolution layers except the last layer. The weights were updated by using random gradient descent (SGD). Finally, the fine-tuned VGG16 yielded 0.97 accuracy and 0.98 AUC.

Kumar et al. [154] proposed a self-made CNN model for breast cancer classification. Six convolutional layers, six max-pooling layers, and two fully connected layers are used to form the self-made CNN model. The ReLU activation function was selected in this paper. The self-made CNN model was tested on the 7909 breast cancer images and achieved 84% efficiency.

Kousalya et al. [155] compared the self-made CNN model with DensenNet201 for the classification of breast cancer. In the self-made CNN model, there were two convolutional layers, two pooling layers, one flattened layer, and two fully connected layers. They tested these two CNN models on the different learning rates and batch sizes. In conclusion, the self-made CNN models with Particle Swarm Optimization (PSO) can yield better specificity and precision.

Mikhailov et al. [156] proposed a novel CNN model to classify breast cancer. In this model, the max-pooling and depth-wise separable convolution were selected to improve the classification performance. What’s more, different activation functions were tested in this paper, which were ReLU, ELU, and Sigmoid. The novel CNN model with ReLU can achieve the best accuracy, which was 85%.

Karthik et al. [157] offered a novel stacking ensemble CNN framework for the classification of breast cancer. Three stacked CNN models were made for extracting features. They designed these three stacked CNN models. The features from these three stacked CNN models were ensembled to yield better classification performance. The ensemble CNN model achieved 92.15 accuracy, 92.21% F1-score, and 92.17% recall.

Nawaz et al. [158] proposed a novel CNN model for the multi-classification of breast cancer. In this model, DenseNet was used as the backbone model. The open data set (BreakHis data set) was selected to test the proposed novel model. The novel model could achieve 95.4% accuracy for the multi-classification of breast cancer.

Deniz et al. [159] proposed a new model for breast cancer classification, which obtained transfer learning and CNN models. The pre-trained VGG16 and AlexNet were used to extract features. These extracted features from these two pre-trained CNN models would be concatenated and then fed to SVM for classification. The model can achieve 91.30% accuracy.

Yeh et al. [160] compared CNN-based CAD and feature-based CAD for classifying breast cancer based on DBT images. In the CNN-based CAD, the feature extractor was the LeNet. After experiments, the LeNet-based CAD could yield 87.12% (0.035) and 74.85% (0.122) accuracy. In conclusion, the CNN-based CAD could outperform the feature-based CAD.

Gonçalves et al. [161] tested three different CNN models to classify breast cancer, which were ResNet50, DenseNet201, and VGG16. In these three CNN models, transfer learning was used to improve classification performance. Finally, the DenseNet could get 91.67% accuracy, 83.3% specificity, 100% sensitivity, and 0.92 F1-score.

Bayramoglu et al. [162] proposed two different CNN models for breast cancer classification. The single CNN model was used to classify a malignancy. The multi-task CNN (mt_CNN) model was used to classify malignancy and image magnification levels. The single CNN model and mt_CNN model could yield 83.25% and 82.13% average recognition rates, respectively.

Alqahtani et al. [163] offered a novel CNN model (msSE-ResNet) for breast cancer classification. In the msSE-ResNet, the residual learning and different scales were used to improve the results. The msSE-ResNet can achieve 88.87% accuracy and 0.9541 AUC.

For the classification of breast cancer based on CNN, there are some limitations. When these existing methods select the large public dataset, it will take a lot of training time. Five-fold cross-validation was used to evaluate some proposed methods in these papers. Even though some results were very good, there were still many unsatisfactory results. The details of these methods are given in Table 6.

### 4.2 Breast Cancer Detection

We will review the detection of breast cancer based on CNN in this section [164]. Researchers use the CNN model to detect candidate lesion locations in breast images.

Sohail et al. [165] introduced a CNN-based framework (MP-MitDet) for mitotic nuclei recognition in pathological images of breast cancer. The framework can be divided into four steps. 1. refine the label, 2 Select split region, 3 Blob analysis, 4 cell refinement. The whole framework used an automatic tagger and the CNN model for training. More areas were selected according to the spot area. The MP-MitDet obtained 0.71 precision, 0.76 recall, 0.75 F1, and 0.78 area.

**Figure 14:**
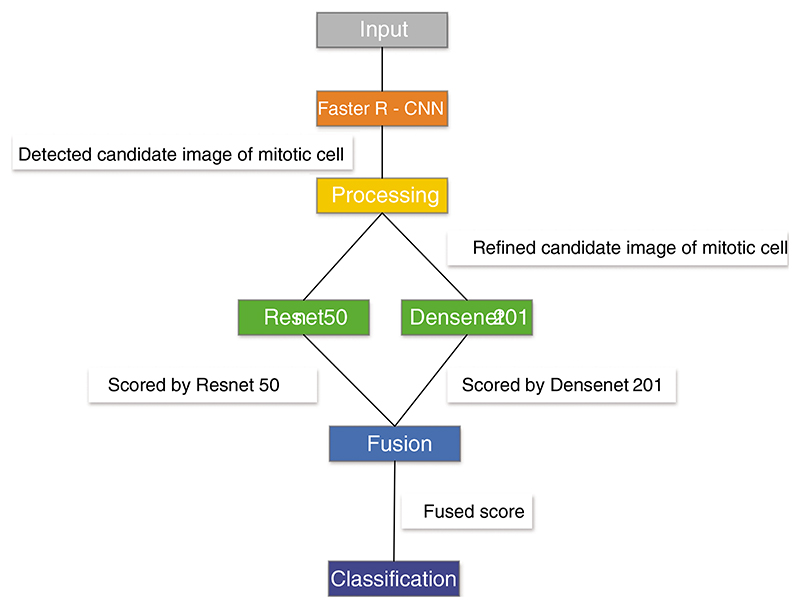
The framework of R-CNN+CNN

Mahmood et al. [166] proposed a low-cost CNN framework for automatic breast cancer mitotic cell detection, as shown in Fig. 14. This framework was composed of the faster regional convolutional neural network (Faster R-CNN) and deep CNN. They experimented with this model on two public datasets, which were ICPR 2012 and ICPR 2014. This model yielded 0.841 recall, 0.858 F1, and 0.876 precision for ICPR 2012 and 0.583 recall, 0.691 F1, and 0.848 precision for ICPR 2014.

Wang et al. [167] introduced a new model by CNN and US-ELM (CNN-GTD-ELM) to detect breast cancer X-rays. They designed an 8-layer CNN model for feature extraction of input images. They combined the extracted features with some additional features of the tumor. These combined features were the input to the ELM.

**Figure 15:**
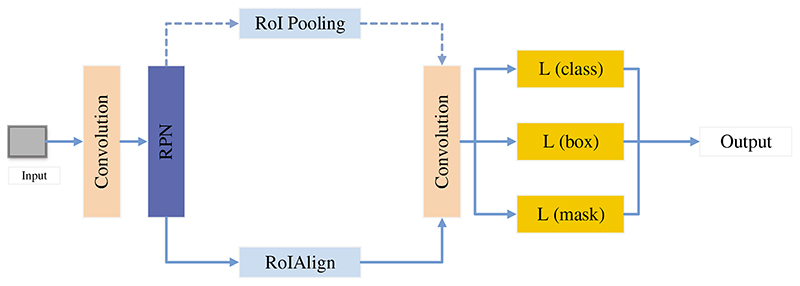
The structure of mask CNN

Chiao et al. [168] established a mask region detection framework based on CNN, as given in Fig. 15. This method detected the lesion of breast cancer and classify benign and malignant breast cancer. Finally, this framework achieved 0.75 average precision in detection and 85% accuracy in classification.

Das et al. [169] introduced Deep Multiple Instance Learning (MIL) based on CNN for breast cancer detection. This model did not rely on region learning marked by experts on WSI images. The MIL-CNN model achieved 96.63%, 93.06%, and 95.83% accuracy on the IUPHL, BreakHis, and UCSB data sets, respectively.

Melekoodappattu et al. [11] introduced a framework for breast cancer detection. The framework was mainly composed of CNN and image texture attribute extraction. They designed a 9-layer CNN model. In the extraction phase, they defined texture features and used Uniform Manifold Approximation and Projection (UMAP) to reduce the dimension of features. Then the multi-stage features were integrated for detection. They tested this model on two data sets which were MIAS and DDSM. This model obtained 98% accuracy and 97.8% specificity for the MIAS data set, and 97.9% accuracy and 98.3% specificity for the DDSM data set.

Zainudin et al. [170] designed three CNN models for mitosis and amitosis in breast cell detection. The layers of these three CNN were 6, 13, and 17, respectively. Experiments showed that the 17-layer CNN model achieved the best performance. Finally, the model achieved a 15.50% loss, 80.55% TPR, 84.49% accuracy, and 11.66% FNR.

Wu et al. [171] introduced a deep fused fully convolutional neural network (FF-CNN) for breast cancer detection. They selected the AlexNet model as the backbone model. They combined different levels of features to improve detection results. They used a multi-step fine-tuning method to reduce overfitting problems. The FF-CNN was tested on ICPR 2014 data set and obtained better detection accuracy and faster detection speed.

Gonçalves et al. [172] introduced a new framework for breast cancer detection. This new framework used two bionic optimization techniques to optimize the CNN model, which were particle swarm optimization and genetic algorithm. The authors used three CNN models, which were DenseNet-201, VGG-16, and ResNet-50. Experiments showed that the optimized network detection results were significantly improved. The F1 score of VGG-16 was increased from 0.66 to 0.92 and the F1 score of ResNet-50 was increased from 0.83 to 0.90. The F1 values of the three optimized networks were higher than 0.90.

Guan et al. [173] proposed two models to detect breast cancer. The first method was to train images by Generative Adversarial Network (GAN) and then put the trained images into CNN for experiments. The accuracy of this model was 98.85%. The second model was that they first select the VGG-16 model as the backbone model and then transferred the backbone model. The accuracy of this method was 91.48%. The authors combined the two methods, but the results of the combined model were not ideal.

Hadush et al. [174] proposed the breast mass abnormality detection model with CNN to reduce the artificial cost. Extracting features was completed by CNN. Then these features were input into the Region Proposed Network (RPN) and Region of Interest (ROI) of fast R-CNN for detection. Finally, the method achieved 92.2% AUC-ROC, 91.86% accuracy, and 94.67% sensitivity.

Huang et al. [175] presented a lightweight CNN model (BM-Net) to detect breast cancer. The lightweight CNN model consisted of MobileNet-V3 and bilinear structure. The MobileNet-V3 was the backbone model to extract the features. To save resources, they just replaced the fully connected layer with a bilinear structure. The BM-Net could achieve 0.88 accuracy and 0.71 score.

Mahbub et al. [176] proposed a novel model to detect breast cancer. They designed a CNN model, which consisted of six convolutional layers, five max-pooling layers, and two dense layers. The proposed model was composed of the designed CNN model and the fuzzy analytical hierarchy process model. The proposed model can get 98.75% accuracy to detect breast cancer.

Prajoth SenthilKumar et al. [177] used a pre-trained CNN model for the detection and analysis of breast cancer. They selected the VGG16 model as the backbone model. They detected breast cancer from the histology images based on the variability, cell density, and tissue structure. The model could get 88% accuracy.

Charan et al. [178] designed a 16-layers CNN model for the detection of breast cancer. The designed CNN model consisted of six convolution layers, four average-pooling layers, and one fully connected layer. The public data set (Mammograms-MIAS data set) was used for training and testing. The designed CNN model can achieve 65% accuracy.

Alanazi et al. [179] offered a novel CNN model for the detection of breast cancer. They designed a new CNN model and used three different classifiers to detect breast cancer. Three classifiers were K-nearest neighbor, logistic regression, and support vector machines, respectively. This new model can achieve 87% accuracy, which improved 9% accuracy than other ML methods.

Gonçalves et al. [180] presented a novel model to detect breast cancer. They proposed a new random forest surrogate to get better parameters in the pre-trained CNN models. The random forest surrogate was made of particle swarm optimization and genetic algorithms. Three pre-trained CNN models were used in this paper, which was ResNet50, DenseNet201, and VGG16. With the help of the proposed random forest surrogate, the F1-scores of DenseNet201 and ResNet50 could be improved from 0.92 to 1, and 0.85 to 0.92, respectively.

Guan et al. [181] applied the generative adversarial network (GAN) to generate more breast cancer images. The regions of interest (ROIs) form images to train GAN. Some augmentation methods were used to compare with GAN, such as scaling, shifting, rotation, and so on. They designed a new CNN model as the classifier. After experiments, the GAN can yield around 3.6% better than other transformations on the image augmentation.

Sun et al. [182] were inspired by human detection to propose a novel model for breast cancer detection based on the mammographic image. The mathematical morphology method was used to preprocess the images. The image template matching method was selected to locate the suspected regions of a breast mass. The PSO was used to improve the accuracy. The proposed model can achieve 85.82% accuracy, 66.31% F1-score, 95.38% recall, and 50.81% precision.

Chauhan et al. [183] used different algorithms to detect breast cancer. Three different algorithms were CNN, KNN, and SVM, respectively. They compared these three algorithms on the breast cancer data set. SVM could achieve 98% accuracy, KNN can yield 73% accuracy, and CNN could get 95% accuracy.

Gupta et al. [184] proposed a modified CNN model for the detection of breast cancer. The backbone of this model was ResNet. They modified the ResNet in three steps. Firstly, they used the dropout of 0.5. Then, the adaptive average pooling and adaptive max pooling were used by two layers of BN, the dropout, and the fully connected layer. The third step was the stride for down-sampling at 3 × 3 convolution. The modified CNN model could achieve 99.75% accuracy, 99.18% precision, and 99.37% recall, respectively.

Chouhan et al. [185] designed a novel framework (DFeBCD) for detecting breast cancer. In the DFeBCD, they designed the highway network based on CNN to select features. There were two classifiers, which were SVM and Emotional Learning inspired Ensemble Classifier (ELiEC). These two classifiers were trained by the selected features. This framework was evaluated by five-fold cross-validation and achieved 80.5% accuracy.

There are some limitations in the detection of breast cancer based on CNN. If the dataset used in the research paper is very large, a sea of computation and time is needed to complete the training. On the other hand, if the dataset used in the research paper is very small, it could cause an overfitting problem. Most of the breast cancer diagnosis model based on CNN uses the pre-trained CNN model to extract features. But at this time, which layer has the best feature? Which layer of features should we extract? The summary of CNN for breast cancer detection is shown in Table 7.

### 4.3 Breast Cancer Segmentation

In this chapter, we will review the segmentation of breast cancer based on CNN. The abnormal areas in breast images would be segmented based on the CNN model. Breast cancer image segmentation compares the similarity of feature factors between images and divides the image into several regions. Breast segmentation involves the removal of background region, pectoral muscles, labels, artifacts, and other defects add during image acquisition. The segmented area could be compared with the manually segmented area to verify the accuracy of the segmentation method.

Chen et al. [186] introduced a new model for the segmentation of breast cancer. This new framework mainly consisted of two steps. The first step was the segmentation CNN model. Another part was the structure of the QA network based on the ResNet-101 model. A structure was used to predict the quality of each slice. Another structure gave the DSC value.

Tsochatzidis et al. [6] introduced a new CNN model to segment breast masses. In this new CNN model, the convolution layer of each layer was modified. The loss function was also modified by adding an extra term. They evaluated the method on DDSM-400 and CBIS-DDSM datasets.

Lei et al. [56] developed a mask score region based on the R-CNN to segment breast tumors. The network consisted of five parts, namely, the regional suggestion network, the mask terminal, the backbone network, the mask scoring header, and the regional convolution neural network header. In this R-CNN model, the region of interest (ROI) was segmented by using the network blocks between module quality and region categories to build a direct correlation integration.

**Figure 16:**
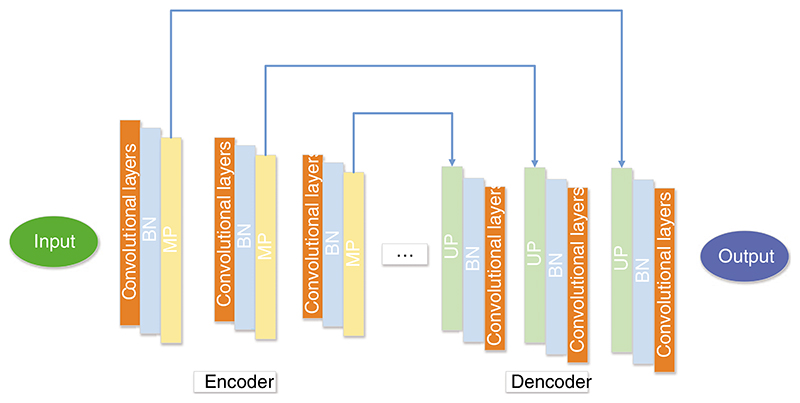
The structure of SegNet

El Adoui et al. [187] proposed two CNN models to segment breast tumors in dynamic contrast-enhanced magnetic resonance imaging (DCE-MRI). The first CNN model was based on SegNet, as presented in Fig. 16. The second model was to select U-Net as the backbone model. 85% of the data sets were used for training, and the other 15% were used for validation. The first method obtained 68.88% IoU, and the second method obtained 76.14% IoU.

Kakileti et al. [188] introduced a cascaded CNN architecture for breast cancer segmentation. This new model used a 5-stage V-net as the main encoding and decoding structure. To improve the accuracy, they used stridden convolutions, deconvolutions, and PReLU activation in this model. This new method obtained 91.6% overall Dice, 93.3% frontal Dice, 89.5% lateral Dice, and 91.9% oblique Dice.

Kumar et al. [189] introduced a dual-layered CNN model (DL-CNN) for breast cancer region recognition and segmentation. The first layer CNN was used to identify the possible region. The second layer CNN was used to segment and reduce false positive. They tested the model on breast image data sets and obtained 0.9726 at 0.39706 for True Positive Rate at False-positive per image.

**Figure 17:**
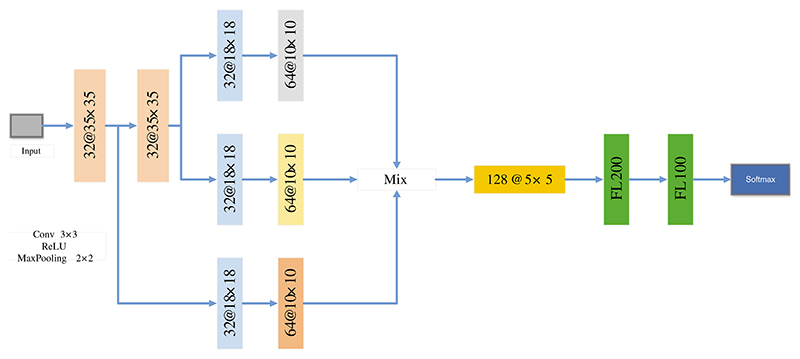
The framework of MRFE-CNN

Ranjbarzadeh et al. [90] proposed a new CNN with multiple feature extraction paths for the segmentation of breast cancer (MRFE-CNN), as shown in Fig. 17. To prevent deep structure, they enhanced the data set. This method can improve the detection of breast cancer tumor boundaries. They used Mini-MIAS and DDSM data sets to evaluate the MRFE-CNN. They obtained 0.936, 0.890, and 0.871 accuracy for normal, benign, and malignant tumors on Mini-MIAS, and 0.944, 0.915, 0.892 accuracy for normal, benign, and malignant tumors on DDSM.

Atrey et al. [190] proposed a new CNN automatic segmentation system for breast lesions. This system was mainly based on their self-made CNN model. For the evaluation of this model, the authors used the bimodal database for bimodal evaluation. The two databases were MG and US. Finally, this model got 0.64 DCS, 0.53 JI for the MG, and 0.77 DSC, 0.64 JI for the US.

Irfan et al. [191] introduced a segmentation model with Dilated Semantic Segmentation Network (Di-CNN) for ultrasonic breast lesion images. This model was mainly composed of two CNN models. A CNN model was DenseNet201 for transfer learning. The second model is a self-made 24-layer CNN model. The features extracted from the two CNN models were fused. SVM was used as the classifier of this model. This model yielded 98.9% accuracy.

Su et al. [192] designed a fast-scanning depth convolution neural network (FCNN) for breast cancer segmentation. This model reduced the amount of calculation and the calculation time. It only took 2.3 s to split 1000 × 1000 images. The FCNN model got 0.91 precision, 0.82 recall, and 0.85 F1.

He et al. [193] proposed a novel network with the CNN model and transferring learning to classify and segment breast cancer. In this paper, two CNN models (AlexNet and GoogleNet) were selected as the backbone models. These two CNN models were used as the feature extractors and SVM was selected as the classifier. The segmentation of this model in breast cancer was similar to professional pathologists.

Soltani et al. [194] introduced a new model for automatic breast cancer segmentation. This method was based on the Mask RCNN. The backbone model used in this paper was detectron2. The model was tested on the INbreast data set and got 81.05% F1 and 95.87% precision.

Min et al. [195] introduced a new system (fully integrated CAD) for the automatic segmentation of breast cancer. The new system was composed of the detection-segmentation method and pseudo-color image generation. The detection-segmentation method was mainly with Mask RCNN. The public INbreast data set was chosen to test the new system. This system yielded a 0.88 Dice similarity index.

Arora et al. [196] proposed a model (RGU-Net) for breast cancer segmentation. The RGU-Net consisted of residual connection and group convolution in U-Net. There were several different sizes of encoder and decoder blocks. The conditional random field was selected to analyze the boundaries. The model was evaluated on the INbreast data set and produced 92.6% Dice.

Spuhler et al. [197] introduced a new CNN method (DCE-MRI) to segment breast cancer. The manual regions of interest were completed by the expert radiologist (R1). R2 and R3 were finished by a resident and another expert radiologist. Finally, the new model 0.71 Dice by using R1.

Atrey et al. [198] proposed a customized CNN for the segmentation of breast cancer based on MG and US. There were nine layers in this customized CNN model. Two convolutional layers, one max-pooling layer, one ReLU layer, one fully connected layer, one softmax layer, and a classification layer formed the whole customized CNN model. This model achieved 0.64 DSC and 0.53 JI for MG and 0.77 DSC and 0.64 JI for the US.

Sumathi et al. [199] proposed a new system to segment breast cancer. They used artificial bee colony optimization with fuzzy clustering to select features. Then, CNN was used as the classifier. This hybrid system could achieve 98% segmentation accuracy.

Xu et al. [200] designed an 8-layer CNN for the segmentation of breast cancer. This customized 8-layer CNN model consisted of 1–3 convolution layers, 1–3 pooling layers, a fully connected layer, and a softmax layer. This customized CNN model yielded 85.1% JSI.

Guo et al. [201] proposed a novel network to segment breast cancer. They designed a 6-layers CNN model, which consisted of two convolutional layers, two pooling layers, and two fully connected layers. The features were extracted by the customized CNN model and then fed to SVM. The proposed combined CNN-SVM achieved 0.92, 0.93, and 0.95 on the sensitivity index, DSC coefficient, and PPV.

Cui et al. [202] proposed a novel patch-based CNN model for the detection of breast cancer based on MRI. They designed a 7-layer CNN model, which consisted of four convolutional layers, two max-pooling layers, and one fully connected layer. The 7-layer CNN model achieved a 95.19% Dice ratio.

For the segmentation of breast cancer based on CNN, there are some limitations. These methods selected public datasets for experiments. But these public datasets need many expert doctors to label these images. What’s more, the application of unsupervised learning technology in the segmentation of breast cancer is not very good. The summary of CNN for breast cancer segmentation is shown in Table 8.

## 5 Conclusion

Recently, the diagnosis of breast cancer based on CNN has made rapid progress and success. This also makes more and more researchers devote more energy to a breast cancer diagnosis with CNN. We complete a comprehensive review of the diagnosis of breast cancer based on CNN after reviewing a sea of recent papers. In this paper, readers can not only see the CNN-based diagnostic methods for breast cancer in recent decades but also know the advantages and disadvantages of these methods and future research directions. The main contributions of this survey: (i) A sea of major papers about the diagnosis of breast cancer based on CNN is reviewed in this paper to provide a comprehensive survey; (ii) This survey presents the advantages and disadvantages of these state-of-the-art methods; (iii) A presentation of significant findings gives readers the opportunities available in the research interest; (iv) We give the future research direction and critical challenges about the CNN-based diagnostic methods for breast cancer.

Based on the papers we have reviewed, many techniques have been used to boost their proposed CNN models for the diagnosis of breast cancer. Many researchers used pre-trained CNN models or their customized CNN models to extract features from input. To reduce the training time and computational cost, some researchers replace some last layers of CNN models with other techniques, such as SVM, ELM, and so on. In some papers, researchers would select more than one CNN models to extract different features. Then, these different features would be ensembled and fed to classifiers for improving performance.

Although this breast cancer diagnosis with CNN has achieved great success, there are still some limitations. (i) There are too few good data sets. A good public breast cancer dataset needs to involve many aspects, such as professional medical knowledge, privacy issues, financial issues, dataset size, and so on. (ii) When the data set is too large, the CNN-based model needs a sea of computation and time to complete the detection. (iii) It is easy to cause overfitting when using small data sets. (iv) Most of the breast cancer diagnosis model based on CNN uses the pre-trained CNN model to extract features. But at this time, which layer has the best feature? Which layer of features should we extract? These problems have not been well solved in recent studies.

Even though this paper reviews a sea of recent research papers, there are still some limitations. First, this survey only pays attention to CNN for breast cancer diagnosis. There are some other CAD methods for breast cancer diagnosis. Second, this survey only focuses on two-dimensional images.

In the future, researchers can try more unlabeled data sets for breast cancer detection. Compared with labeled datasets, unlabeled datasets are less expensive and more numerous. What’s more, researchers can try more new methods for image feature extraction, such as EL, TL, xDNNs, U-Net, transformer, and so on.

This paper reviews the CNN-based breast cancer diagnosis technology in recent years. With the progress of CNN technology, the diagnosis accuracy of breast cancer is getting higher and higher. We summarize the limitations and future research directions of CNN-based breast cancer diagnosis technology. Although breast cancer diagnosis technology based on CNN has achieved great success and can be used as an auxiliary means to help doctors diagnose breast cancer, there is still much to be improved.

## Figures and Tables

**Table 1:** Full explanation and abbreviated imaging modality

Abbreviated imaging modality	Full explanation
SFM	Screen-film mammography
DM	Digital mammography
MRI	Magnetic resource imaging
US	Ultrasound
DBT	Digital breast tomosynthesis
HP	Histopathological images

**Table 2:** Advantages and disadvantages of abbreviated imaging modality

Abbreviated imaging modality	Advantages	Disadvantages
SFM	Detect the early-stage cancer Standard imaging modality High sensitivity	Not digital imaging modality Low sensitivity with dense cancer Image is impossible to be improved
DM	Effective imaging modality Detects the early-stage breast cancer	Expensive compared with SFM High radiation exposure Low specificity may cause unnecessary biopsies High false-positive results and false-negative results
MRI	Used for clinical diagnosis Suitable for high-risk patients High sensitivity	Low specificity High cost compared with US and DM
US	No radiation Suitable for pregnant patients Safe and low cost compared with SFM and DM	High requirement for operator
DBT	High accuracy, sensitivity, and specificity compared with DM Less time for imaging More details of the dense chest Multiple 3D images	High cost compared with the other four imaging modalities Not detect malignant micro-calcifications
HP	Get depth information Better resolution Capture cell shape information Capture structural information	Invasive Require additional costs

**Table 3:** The characteristics of activation functions

Activation function	Symmetry the origin	Speed of convergence	Output
Sigmoid	No	low	(0, 1)
Tanh	Symmetrical	low	(−1, 1)
ReLU	No	Fast	[0, +∞)
LReLU	No	Fast	(−∞, +∞)
PReLU	No	Fast	(−∞, +∞)

**Table 4:** Confusion matrix

	Predicated class	
True class	TP	FN
	FP	TN

**Table 5:** The details of some public data sets

Date set	Number of images	Size (GB)	Modality
DDSM	55,890	-	DM
MIAS	322	2.3	DM
CBIS-DDSM	4067	70.5	DM
INbreast	410	-	DM
BreakHis	9109	-	Histology

Note: - means unavailable.

**Table 6:** Details of breast cancer classification based on CNN

Authors	Methods	Results
Alkhaleefah et al. [141]	A model combining CNN and SVM classifier with RBF to classify breast cancer.	The sensitivity, specificity, and accuracy of this model were 1, 0.86, and 0.92, respectively.
Liu et al. [142]	The fully connected layer first CNN (FCLF-CNN) method was proposed. This method added the fully connected layer before the convolution layer. They improved structured data transformation in two ways. The encoder in the first method was the fully connected layer. The second method was to use MSE losses.	The FCLF-CNN model got 99.28% accuracy, 98.65% sensitivity, and 99.57% specificity for the WDBC data set, and 98.71% accuracy, 97.60% sensitivity, and 99.43% specificity for the WBCD data set.
Gour et al. [143]	A network (ResHist) was designed to classify breast cancer. The data enhancement technique combined affine transformation, stain normalization, and image patch generation.	This method finally achieved 84.34% accuracy and 90.49% F1.
Wang et al. [144]	A hybrid CNN and SVM model was presented to classify breast cancer. This method uses the VGG16 network as the backbone model.	The accuracy of this method was 80.6%.
Yao et al. [145]	This model used CNN (DenseNet) and RNN (LSTM) to extract features. Then the perceptron attention mechanism based on NLP was used to weigh the features.	This model achieved 98.3% accuracy, 100% precision, 100% recall, 100% F1 for Bioimaging2015 Dataset.
Ibraheem et al. [24]	A three-parallel CNN branch network (3PCNNB-Net) was designed to classify breast cancer. There were three parallel CNN to extract the features. Then, they used the average layer to merge the features. The f lattened layer, BN, and softmax layer were used as the classification layer.	The 3PCNNB-Net achieved 97.04% accuracy, 97.14% sensitivity, and 95.23% specificity.
Agnes et al. [146]	A multiscale all convolutional neural network (MA-CNN) was proposed for breast cancer classification. In the MA-CNN, they used extended convolution and used three dilated convolutions of different sizes to extract different levels’ features.	The accuracy, sensitivity, specificity, F1, and AUC of MA-CNN were 96.47%, 96%, 96%, 96%, and 0.99, respectively.
Zhang et al. [115]	An 8-layer CNN network (BDR-CNN-GCN) was designed for breast cancer classification. They integrated BN and dropout, replaced the normal pooling layer with RSP, and combined GCN.	The accuracy, sensitivity, and specificity of BDR-CNN-GCN were 96.10%±1.60%, 96.20%±2.90%, and 96.00%±2.31%, respectively.
Wang et al. [147]	A breast cancer classification model based on CNN (inception-v3) was proposed.	This model got 0.886 sensitivity, 0.876 specificity, and 0.9468 AUC, respectively.
Saikia et al. [148]	They compared different classical CNN models in breast cancer classification, which were VGG16, VGG19, ResNet-50, and GoogLeNet-V3.	Finally, GoogLeNet-V3 achieved the highest accuracy of 96.25%.
Mewada et al. [149]	A new CNN-based model was proposed to classify breast cancer. In this new model, they added the multi-resolution wavelet transform.	They tested the new model on the BreakHist dataset and BCC2015 and obtained 97.58% and 97.45% accuracy, respectively.
Zhou et al. [150]	A new model was proposed for automatically classifying benign and malignant breast cancer, which combined SWE and the CNN model.	This SWE-CNN model produced 95.7% specificity, 96.2% sensitivity, and 95.8% accuracy, respectively.
Lotter et al. [151]	A multi-scale CNN was designed for the classification of breast cancer.	They tested the multi-scale CNN on the DDSM dataset and obtained 0.92 AUROC.
Vidyarthi et al. [152]	A classification method combining CLAHE, and CNN model was proposed for microscopic imaging of breast cancer.	The results showed that the hybrid model of CNN can get better classification results, which produces an accuracy of about 90%.
Hijab et al. [153]	A classical CNN model (VGG16) was used for breast cancer classification. They did some modifications to the VGG16.	Finally, the fine-tuned VGG16 yielded 0.97 accuracy and 0.98 AUC.
Kumar et al. [154]	Six convolutional layers, six max-pooling layers, and two fully connected layers are used to form the self-made CNN model.	The self-made CNN model was tested on the 7909 breast cancer images and achieved 84% efficiency.
Kousalya et al. [155]	The self-made CNN model was compared with DensenNet201 for the classification of breast cancer. These two CNN models were tested on different learning rates and batch sizes.	The self-made CNN models with Particle Swarm Optimization (PSO) can yield better specificity and precision.
Mikhailov et al. [156]	The max-pooling and depth-wise separable convolution were used in this novel CNN model to classify breast cancer. ReLU, ELU, and Sigmoid were tested in this paper.	The novel CNN model with ReLU can achieve 85% accuracy.
Karthik et al. [157]	A novel stacking ensemble CNN framework was proposed for the classification of breast cancer. They designed these three stacked CNN models for extracting features. These extracted features were ensembled for classification.	The ensemble CNN model achieved 92.15 accuracy, 92.21% F1-score, and 92.17% recall.
Nawaz et al. [158]	A novel CNN model was proposed for the multi-classification of breast cancer. In this model, DenseNet was used as the backbone model.	The novel model could achieve 95.4% accuracy for the multi-classification of breast cancer.
Deniz et al. [159]	A new model was based on obtained transfer learning and CNN models. for breast cancer classification. The pre-trained VGG16 and AlexNet were used to extract features. These extracted features would be concatenated and then fed to SVM for classification.	The model could achieve 91.30% accuracy.
Yeh et al. [160]	The CNN-based CAD and feature-based CAD for classifying breast cancer were compared. In the CNN-based CAD, the feature extractor was the LeNet.	In conclusion, the CNN-based CAD could outperform the feature-based CAD.
Gonçalves et al. [161]	Three different CNN models were tested to classify breast cancer, which were ResNet50, DenseNet201, and VGG16.	DenseNet could achieve the best results and get 91.67% accuracy, 83.3% specificity, 100% sensitivity, and 0.92 F1-score.
Bayramoglu et al. [162]	Two different CNN models were proposed for breast cancer classification. The single CNN model was used to classify a malignancy. The multi-task CNN (mt_CNN) model was used to classify malignancy and image magnification levels.	The single CNN model and mt_CNN model could yield 83.25% and 82.13% average recognition rates, respectively.
Alqahtani et al. [163]	A novel CNN model (msSE-ResNet) for breast cancer classification. In the msSE-ResNet, the residual learning and different scales were used to improve the results.	The msSE-ResNet can achieve 88.87% accuracy and 0.9541 AUC.

**Table 7:** Summary of CNN for breast cancer detection

Authors	Methods	Results
Sohail et al. [165]	A CNN-based framework (MP-MitDet) was proposed for mitotic nuclei recognition in pathological images of breast cancer. The whole framework used an automatic tagger and the CNN model for training.	The MP-MitDet obtained 0.71 precision, 0.76 recall, 0.75 F1, and 0.78 area.
Mahmood et al. [166]	A low-cost CNN-based model was proposed for automatic breast cancer mitotic cell detection. This framework was composed of the faster regional convolutional neural network (Faster R-CNN) and deep CNN.	This model yielded 0.841 recall, 0.858 F1, and 0.876 precision for ICPR 2012 and 0.583 recall, 0.691 F1, and 0.848 precision for ICPR 2014.
Wang et al. [167]	A new model combining CNN and US-ELM (CNN-GTD-ELM) was proposed to detect breast cancer X-rays. They designed an 8-layer CNN model for feature extraction of input images and used ELM for detection. They combined the extracted features with some additional features of the tumor.	The CNN-GTD-ELM got 86.50% accuracy, 85.10% sensitivity, 88.02% specificity, and 0.923 AUC.
Chiao et al. [168]	A mask region detection method was established based on CNN. This method detected the lesion of breast cancer based on ultrasound images.	Finally, this method achieved 0.75 average precision in detection and 85% accuracy in classification.
Das et al. [169]	A Deep Multiple Instance Learning (MIL) was designed based on the CNN model for breast cancer detection.	The MIL-CNN model achieved 96.63%, 93.06%, and 95.83% accuracy on the IUPHL, BreakHis, and UCSB data sets, respectively.
Melekoodappattu et al. [11]	They proposed the 9-layer CNN method to detect breast cancer. Then, they defined texture features and used Uniform Manifold Approximation and Projection (UMAP) to reduce the dimension of features. The multi-stage features were integrated for detection.	This model obtained 98% accuracy and 97.8% specificity for the MIAS data set, and 97.9% accuracy and 98.3% specificity for the DDSM data set.
Zainudin et al. [170]	They designed three CNN models for mitosis and amitosis in breast cell detection. The layers of these three CNN were 6, 13, and 17, respectively.	Experiments showed that the 17-layer CNN model achieved the best results. Finally, the model achieved a 15.50% loss, 80.55% TPR, 84.49% accuracy, and 11.66% FNR.
Wu et al. [171]	A deep fused fully convolutional neural network (FF-CNN) was designed for breast cancer detection. They selected the AlexNet model as the backbone model and combined different levels of features.	The FF-CNN was tested on ICPR 2014 data set and obtained better detection accuracy and faster detection speed.
Gonçalves et al. [172]	This new framework used particle swarm optimization and genetic algorithm to optimize the CNN model. DenseNet-201, VGG-16, and ResNet-50 were used as the backbone model.	The F1 score of VGG-16 was increased from 0.66 to 0.92 and the F1 score of ResNet-50 was increased from 0.83 to 0.90. The F1 values of the three optimized networks were higher than 0.90.
Guan et al. [173]	Two methods were proposed to detect breast cancer. The first method was to train images by Generative Adversarial Network (GAN) and then put the trained images into CNN for experiments. The second method was that they first select the VGG-16 model as the backbone model and then transferred the backbone model.	The accuracy of the first and second methods were 98.85% and 91.48%.
Hadush et al. [174]	Extracting features was completed by CNN. Then these features were input into the Region Proposed Network (RPN) and Region of Interest (ROI) of fast R-CNN for detection.	The method achieved 92.2% AUC-ROC, 91.86% accuracy, and 94.67% sensitivity.
Huang et al. [175]	A lightweight CNN model (BM-Net) was presented to detect breast cancer. The lightweight CNN model consisted of MobileNet-V3 and bilinear structure. The MobileNet-V3 was the backbone model to extract the features. To save resources, they just replaced the fully connected layer with a bilinear structure.	The BM-Net could achieve 0.88 accuracy and 0.71 score.
Mahbub et al. [176]	The proposed model was composed of the designed CNN model and the fuzzy analytical hierarchy process model. The designed CNN model consisted of six convolutional layers, five max-pooling layers, and two dense layers.	The proposed model can get 98.75% accuracy to detect breast cancer.
Prajoth SenthilKumar et al. [177]	The VGG16 model was selected for the detection and analysis of breast cancer. They detected breast cancer from the histology images based on the variability, cell density, and tissue structure.	The model could get 88% accuracy on the testing data set.
Charan et al. [178]	They designed a 16-layers CNN model for the detection of breast cancer, which consisted of six convolution layers, four average-pooling layers, and one fully connected layer. The public data set (Mammograms-MIAS data set) was used for training and testing.	The designed CNN model can achieve 65% accuracy.
Alanazi et al. [179]	They designed a new CNN model and used three different classifiers to detect breast cancer, which were K-nearest neighbor, logistic regression, and support vector machines, respectively.	This new model can achieve 87% accuracy, which improved 9% accuracy than other ML methods.
Gonçalves et al. [180]	They proposed a new random forest surrogate to get better parameters in the pre-trained CNN models, which were made of particle swarm optimization and genetic algorithms. Three pre-trained CNN models were used in this paper, which were ResNet50, DenseNet201, and VGG16.	With the help of the proposed random forest surrogate, the F1-scores of DenseNet201 and ResNet50 could be improved from 0.92 to 1, and 0.85 to 0.92, respectively.
Guan et al. [181]	The Generative Adversarial Network (GAN) was applied to generate more breast cancer images. The Regions of Interest (ROIs) form images to train GAN. Some augmentation methods were used to compare with GAN, such as scaling, shifting, rotation, and so on. They designed a new CNN model as the classifier.	After experiments, the GAN can yield around 3.6% better than other transformations on the image augmentation.
Sun et al. [182]	A novel model was proposed for breast cancer detection based on the mammographic image. The mathematical morphology method was used to preprocess the images. The image template matching method was selected to locate the suspected regions of a breast mass. The PSO was used to improve the accuracy.	The proposed model can achieve 85.82% accuracy, 66.31% F1-score, 95.38% recall, and 50.81% precision.
Chauhan et al. [183]	Three different algorithms were used to detect breast cancer, which were CNN, KNN, and SVM, respectively.	SVM could achieve 98% accuracy, KNN can yield 73% accuracy, and CNN could get 95% accuracy.
Gupta et al. [184]	A novel modified CNN model was proposed for the detection of breast cancer. They modified the ResNet in three steps. Firstly, they used the dropout of 0.5. Then, the adaptive average pooling and adaptive max pooling were used by two layers of BN, the dropout, and the fully connected layer. The third step was the stride for down-sampling at 3 × 3 convolution.	The modified CNN model could achieve 99.75% accuracy, 99.18% precision, and 99.37% recall, respectively.
Chouhan et al. [185]	A novel framework (DFeBCD) was designed for detecting breast cancer. In the DFeBCD, they designed the highway network based on CNN to select features. There were two classifiers, which were SVM and Emotional Learning inspired Ensemble Classifier (ELiEC). These two classifiers were trained by the selected features.	This framework was evaluated by five-fold cross-validation and achieved 80.5% accuracy.

**Table 8:** Summary of CNN for breast cancer segmentation

Authors	Methods	Results
Chen et al. [186]	A new framework was introduced for the segmentation of breast cancer. This new framework mainly consisted of two parts, which were the segmentation CNN model, and the structure of the QA network based on the ResNet-101 model.	The final accuracy of this method was 0.97, 0.94, and 0.89 respectively; the F1 was 0.98, 0.91, and 0.81 respectively; AUC was 0.96, 0.93, and 0.88 for good, medium, and poor-quality slices, respectively and 0.06 ± 0.19 MAE.
Tsochatzidis et al. [6]	A new CNN model was introduced to segment breast masses. In this new CNN model, the convolution layer of each layer and the loss function were modified.	The AUC of this method was 0.898 and 0.862 for DDSM-400 and CBIS-DDSM, respectively.
Lei et al. [56]	A mask score region based on the R-CNN was proposed to segment breast tumors. The network consisted of five parts, namely, the regional suggestion network, the mask terminal, the backbone network, the mask scoring header, and the regional convolution neural network header.	The R-CNN produced HD95, MSD, RMSD and CMD of 1.646±1.191 mm and 1.665±1.129 mm, 0.489±0.406 mm and 0.475±0.371 mm, 0.755±0.755 mm and 0.751±0.508 mm, 0.672±0.612 mm and 0.665±0.729 mm in two tests, respectively.
El Adoui et al. [187]	Two CNN models were proposed to segment breast tumors in DCE-MRI. The first CNN model was based on SegNet. The second model was to select U-Net as the backbone model.	The first method obtained 68.88% IoU, and the second method obtained 76.14% IoU.
Kakileti et al. [188]	The new model used a 5-stage V-net as the main encoding and decoding structure proposed to segment breast cancer.	This new method obtained 91.6% overall Dice, 93.3% frontal Dice, 89.5% lateral Dice, and 91.9% oblique Dice.
Kumar et al. [189]	A dual-layered CNN model (DL-CNN) was proposed for breast cancer region recognition and segmentation. The first layer CNN was used to identify the possible region. The second layer CNN was used to segment and reduce false positive.	They tested the model on breast image data sets and obtained 0.9726 at 0.39706 for True Positive Rate at False-positive per image.
Ranjbarzadeh et al. [90]	A shallow convolutional neural network with multiple feature extraction paths was proposed for the automatic segmentation of breast cancer (MRFE-CNN).	They obtained 0.936, 0.890, and 0.871 accuracy for normal, benign, and malignant tumors on Mini-MIAS, and 0.944, 0.915, 0.892 accuracy for normal, benign, and malignant tumors on DDSM.
Atrey et al. [190]	A new computer-aided automatic segmentation system was designed for breast lesions, which was mainly based on their self-made CNN model.	This model got 0.64 DSC, 0.53 JI for the MG, and 0.77 DSC, 0.64 JI for the US.
Irfan et al. [191]	Two CNN models were proposed to segment breast lesion images, which were DenseNet201 and a self-made 24-layer CNN model.	This model yielded 98.9% accuracy.
Su et al. [192]	A fast-scanning depth convolution neural network (FCNN) was designed for breast cancer segmentation.	The FCNN model got 0.91 precision, 0.82 recall, and 0.85 F1.
He et al. [193]	Two CNN models (AlexNet and GoogleNet) were selected as the backbone models to classify and segment breast cancer.	The segmentation of this model in breast cancer was similar to professional pathologists.
Soltani et al. [194]	A new method was designed for breast cancer segmentation with the Mask RCNN.	The method was tested on the INbreast data set and achieved 81.05% F1 and 95.87% precision.
Min et al. [195]	A new system (fully integrated CAD) was designed for the automatic segmentation of breast cancer, which was composed of the detection-segmentation method and pseudo-color image generation.	This system yielded a 0.88 Dice similarity index.
Arora et al. [196]	A model (RGU-Net) was designed for breast cancer segmentation, which was composed of residual connection and group convolution in U-Net.	The model was evaluated on the INbreast data set and produced 92.6% Dice.
Spuhler et al. [197]	A new CNN model (DCE-MRI) was designed to segment breast cancer.	The new model achieved 0.71 Dice by using R1.
Atrey et al. [198]	A customized CNN was proposed for the segmentation of breast cancer based on MG and US. There were nine layers in this customized CNN model. Two convolutional layers, one max-pooling layer, one ReLU layer, one fully connected layer, one softmax layer, and a classification layer formed the whole customized CNN model.	This model achieved 0.64 DSC and 0.53 JI for MG and 0.77 DSC and 0.64 JI for the US.
Sumathi et al. [199]	A new system was proposed to segment breast cancer. They used artificial bee colony optimization with fuzzy clustering to select features. Then, CNN was used as the classifier.	This hybrid system could achieve 98% segmentation accuracy.
Xu et al. [200]	An 8-layer CNN was designed for the segmentation of breast cancer. This customized 8-layer CNN model consisted of 1–3 convolution layers, 1–3 pooling layers, a fully connected layer, and a softmax layer.	This customized CNN model yielded 85.1% JSI.
Guo et al. [201]	A novel network was proposed to segment breast cancer. They designed a 6-layers CNN model, which consisted of two convolutional layers, two pooling layers, and two fully connected layers. The features were extracted by the customized CNN model and then fed to SVM.	The proposed combined CNN-SVM achieved 0.92, 0.93, and 0.95 on the sensitivity index, DSC coefficient, and PPV.
Cui et al. [202]	A novel patch-based CNN model was proposed for the detection of breast cancer based on MRI. They designed a 7-layer CNN model, which consisted of four convolutional layers, two max-pooling layers, and one fully connected layer.	The 7-layer CNN model achieved a 95.19% Dice ratio.

## References

[1] Bray F, Laversanne M, Weiderpass E, Soerjomataram I (2021). The ever-increasing importance of cancer as a leading cause of premature death worldwide. Cancer.

[2] Desai M, Shah M (2021). An anatomization on breast cancer detection and diagnosis employing multilayer perceptron neural network (MLP) and convolutional neural network (CNN). Clinical eHealth.

[3] Beeravolu AR, Azam S, Jonkman M, Shanmugam B, Kannoorpatti K (2021). Preprocessing of breast cancer images to create datasets for deep-CNN. IEEE Access.

[4] Sung H, Ferlay J, Siegel RL, Laversanne M, Soerjomataram I (2021). Global cancer statistics 2020: GLOBOCAN estimates of incidence and mortality worldwide for 36 cancers in 185 countries. CA: A Cancer Journal for Clinicians.

[5] Heenaye-Mamode Khan M, Boodoo-Jahangeer N, Dullull W, Nathire S, Gao X (2021). Multiclass classification of breast cancer abnormalities using deep convolutional neural network (CNN). PLoS One.

[6] Tsochatzidis L, Koutla P, Costaridou L, Pratikakis I (2021). Integrating segmentation information into CNN for breast cancer diagnosis of mammographic masses. Computer Methods and Programs in Biomedicine.

[7] Xie XZ, Niu JW, Liu XF, Li QF, Wang Y (2022). DG-CNN: Introducing margin information into convolutional neural networks for breast cancer diagnosis in ultrasound images. Journal of Computer Science and Technology.

[8] Waks AG, Winer EP (2019). Breast cancer treatment: A review. Jama.

[9] Zuluaga-Gomez J, Al Masry Z, Benaggoune K, Meraghni S, Zerhouni N (2021). A CNN-based methodology for breast cancer diagnosis using thermal images. Computer Methods in Biomechanics and Biomedical Engineering: Imaging Visualization.

[10] Sannasi Chakravarthy S, Bharanidharan N, Rajaguru H (2022). Multi-deep CNN based experimentations for early diagnosis of breast cancer. IETE Journal of Research.

[11] Melekoodappattu JG, Dhas AS, Kandathil BK, Adarsh K (2022). Breast cancer detection in mammogram: Combining modified CNN and texture feature based approach. Journal of Ambient Intelligence and Humanized Computing.

[12] Lu J, Wu Y, Xiong Y, Zhou Y, Zhao Z (2022). Breast tumor computer-aided detection system based on magnetic resonance imaging using convolutional neural network. Computer Modeling in Engineering Sciences.

[13] Ribli D, Horváth A, Unger Z, Pollner P, Csabai I (2018). Detecting and classifying lesions in mam-mograms with deep learning. Scientific Reports.

[14] Hance KW, Anderson WF, Devesa SS, Young HA, Levine PH (2005). Trends in inflammatory breast carcinoma incidence and survival: The surveillance, epidemiology, and end results program at the National Cancer Institute. Journal of the National Cancer Institute.

[15] Tabar L, Gad A, Holmberg L, Ljungquist U, Group KCP (1985). Reduction in mortality from breast cancer after mass screening with mammography: Randomised trial from the breast cancer screening working group of the Swedish National Board of Health and Welfare. The Lancet.

[16] Sharma GN, Dave R, Sanadya J, Sharma P, Sharma K (2010). Various types and management of breast cancer: An overview. Journal of Advanced Pharmaceutical Technology Research.

[17] McKinney SM, Sieniek M, Godbole V, Godwin J, Antropova N (2020). International evaluation of an AI system for breast cancer screening. Nature.

[18] Zebari DA, Ibrahim DA, Zeebaree DQ, Mohammed MA, Haron H (2021). Breast cancer detection using mammogram images with improved multi-fractal dimension approach and feature fusion. Applied Sciences.

[19] Mihaylov I, Nisheva M, Vassilev D (2018). Machine learning techniques for survival time prediction in breast cancer.

[20] Zhu Z, Harowicz M, Zhang J, Saha A, Grimm LJ (2018). Medical imaging 2018: Computer-aided diagnosis, vol. 10575, 105752W.

[21] Grimm LJ, Ryser MD, Partridge AH, Thompson AM, Thomas JS (2017). Surgical upstaging rates for vacuum assisted biopsy proven DCIS: Implications for active surveillance trials. Annals of Surgical Oncology.

[22] Veta M, Pluim JP, van Diest PJ, Viergever MA (2014). Breast cancer histopathology image analysis: A review.

[23] Zebari DA, Ibrahim DA, Zeebaree DQ, Haron H, Salih MS (2021). Systematic review of computing approaches for breast cancer detection based computer aided diagnosis using mammogram images. Applied Artificial Intelligence.

[24] Ibraheem AM, Rahouma KH, Hamed HF (2021). 3PCNNB-Net: Three parallel CNN branches for breast cancer classification through histopathological images. Journal of Medical and Biological Engineering.

[25] Mokhatri-Hesari P, Montazeri A (2020). Health-related quality of life in breast cancer patients: Review of reviews from 2008 to 2018. Health and Quality of Life Outcomes.

[26] Kösüs N, Kösüs A, Duran M, Simavli S, Turhan N (2010). Comparison of standard mammography with digital mammography and digital infrared thermal imaging for breast cancer screening. Journal ofthe Turkish German Gynecological Association.

[27] Murtaza G, Shuib L, Abdul Wahab AW, Mujtaba G, Nweke HF (2020). Deep learning-based breast cancer classification through medical imaging modalities: State of the art and research challenges. Artificial Intelligence Review.

[28] Debelee TG, Schwenker F, Ibenthal A, Yohannes D (2020). Survey of deep learning in breast cancer image analysis. Evolving Systems.

[29] Liu J, Zarshenas A, Qadir A, Wei Z, Yang L (2018). Medical imaging 2018: Image processing, vol. 10574, 105740F.

[30] Zhao Z, Wu F (2010). Minimally-invasive thermal ablation of early-stage breast cancer: A systemic review. European Journal of Surgical Oncology.

[31] Jalalian A, Mashohor SB, Mahmud HR, Saripan MIB, Ramli ARB (2013). Computer-aided detection/diagnosis of breast cancer in mammography and ultrasound: A review. Clinical Imaging.

[32] Gilbert FJ, Tucker L, Gillan MG, Willsher P, Cooke J (2015). Accuracy of digital breast tomosynthesis for depicting breast cancer subgroups in a UK retrospective reading study (TOMMY trial). Radiology.

[33] Antropova NO, Abe H, Giger ML (2018). Use of clinical MRI maximum intensity projections for improved breast lesion classification with deep convolutional neural networks. Journal of Medical Imaging.

[34] Griebsch I, Brown J, Boggis C, Dixon A, Dixon M (2006). Cost-effectiveness of screening with contrast enhanced magnetic resonance imaging vs X-ray mammography of women at a high familial risk of breast cancer. British Journal of Cancer.

[35] Kuhl CK, Schrading S, Bieling HB, Wardelmann E, Leutner CC (2007). MRI for diagnosis of pure ductal carcinoma in situ: A prospective observational study. The Lancet.

[36] Kelly KM, Dean J, Comulada WS, Lee SJ (2010). Breast cancer detection using automated whole breast ultrasound and mammography in radiographically dense breasts. European Radiology.

[37] Shin SY, Lee S, Yun ID, Kim SM, Lee KM (2018). Joint weakly and semi-supervised deep learning for localization and classification of masses in breast ultrasound images. IEEE Transactions on Medical Imaging.

[38] Byra M, Sznajder T, Korzinek D, Piotrzkowska-Wróblewska H, Dobruch-Sobczak K (2019). Impact of ultrasound image reconstruction method on breast lesion classification with deep learning.

[39] Fotin SV, Yin Y, Haldankar H, Hoffmeister JW, Periaswamy S (2016). Medical imaging 2016: Computer-aided diagnosis.

[40] Zhang J, Ghate SV, Grimm LJ, Saha A, Cain EH (2018). Medical imaging 2018: Computer-aided diagnosis, vol. 10575, 105752V.

[41] Hooley RJ, Durand MA, Philpotts LE (2017). Advances in digital breast tomosynthesis. American Journal of Roentgenology.

[42] Samala RK, Chan HP, Hadjiiski L, Helvie MA, Wei J (2016). Mass detection in digital breast tomosynthesis: Deep convolutional neural network with transfer learning from mammography. Medical Physics.

[43] Pang T, Wong JHD, Ng WL, Chan CS (2020). Deep learning radiomics in breast cancer with different modalities: Overview and future. Expert Systems with Applications.

[44] Mahmood T, Li J, Pei Y, Akhtar F, Imran A (2020). A brief survey on breast cancer diagnostic with deep learning schemes using multi-image modalities. IEEE Access.

[45] Kumar G, Alqahtani H (2022). Deep learning-based cancer detection-recent developments,trend and challenges. Computer Modeling in Engineering Sciences.

[46] Zou L, Yu S, Meng T, Zhang Z, Liang X (2019). A technical review of convolutional neural network-based mammographic breast cancer diagnosis. Computational and Mathematical Methods in Medicine.

[47] Gurcan MN, Boucheron LE, Can A, Madabhushi A, Rajpoot NM (2009). Histopathological image analysis: A review. IEEE Reviews in Biomedical Engineering.

[48] Ertosun MG, Rubin DL (2015). Probabilistic visual search for masses within mammography images using deep learning.

[49] Hussein IJ, Burhanuddin MA, Mohammed MA, Benameur N, Maashi MS (2022). Fully-automatic identification of gynaecological abnormality using a new adaptive frequency filter and histogram of oriented gradients (HOG). Expert Systems.

[50] Tang J, Rangayyan RM, Xu J, El Naqa I, Yang Y (2009). Computer-aided detection and diagnosis of breast cancer with mammography: Recent advances. IEEE Transactions on Information Technology in Biomedicine.

[51] Yassin NI, Omran S, El Houby EM, Allam H (2018). Machine learning techniques for breast cancer computer aided diagnosis using different image modalities: A systematic review. Computer Methods and Programs in Biomedicine.

[52] Xie S, Yu Z, Lv Z (2021). Multi-disease prediction based on deep learning: A survey. Computer Modeling in Engineering and Sciences.

[53] Doi K (2007). Computer-aided diagnosis in medical imaging: Historical review, current status and future potential. Computerized Medical Imaging and Graphics.

[54] Sadaf A, Crystal P, Scaranelo A, Helbich T (2011). Performance of computer-aided detection applied to full-field digital mammography in detection of breast cancers. European Journal of Radiology.

[55] Karimi Jafarbigloo S, Danyali H (2021). Nuclear atypia grading in breast cancer histopathological images based on CNN feature extraction and LSTM classification. CAAI Transactions on Intelligence Technology.

[56] Lei Y, He X, Yao J, Wang T, Wang L (2021). Breast tumor segmentation in 3D automatic breast ultrasound using mask scoring R-CNN. Medical Physics.

[57] Salama WM, Aly MH (2021). Deep learning in mammography images segmentation and classification: Automated CNN approach. Alexandria Engineering Journal.

[58] Agarwal P, Yadav A, Mathur P (2022). Data engineering for smart systems.

[59] Nazir MS, Khan UG, Mohiyuddin A, Reshan A, Saleh M (2022). A novel CNN-inception-V4-based hybrid approach for classification of breast cancer in mammogram images. Wireless Communications and Mobile Computing.

[60] Qin C, Wu Y, Zeng J, Tian L, Zhai Y (2022). Joint transformer and multi-scale CNN for DCE-MRI breast cancer segmentation. Soft Computing.

[61] Zainudin Z, Shamsuddin SM, Hasan S (2021). Machine learning and big data analytics paradigms: Analysis, applications and challenges.

[62] Agarwal R, Sharma H (2021). Advances in computer, communication and computational sciences.

[63] Saber A, Sakr M, Abou-Seida O, Keshk A (2021). A novel transfer-learning model for automatic detection and classification ofbreast cancer based deep CNN. Kafrelsheikh Journal of Information Sciences.

[64] Shaila S, Gurudas V, Hithyshi K, Mahima M, PoojaShree H (2022). Data engineering and intelligent computing.

[65] Karuppasamy A, Abdesselam A, Hedjam R, Zidoum H, Al-Bahri M (2022). Recent CNN-based techniques for breast cancer histology image classification. The Journal of Engineering Research [TJER].

[66] Susilo AB, Sugiharti E (2021). Accuracy enhancement in early detection of breast cancer on mammogram images with convolutional neural network (CNN) methods using data augmentation and transfer learning. Journal of Advances in Information Systems and Technology.

[67] Hariharan R, Dhilsath Fathima M, Pitchai A, Roy VJ, Padhi A (2022). Advance concepts of image processing and pattern recognition.

[68] Bal A, Das M, Satapathy SM, Jena M, Das SK (2021). BFCNet: A CNN for diagnosis of ductal carcinoma in breast from cytology images. Pattern Analysis and Applications.

[69] Kumar A, Sharma A, Bharti V, Singh AK, Singh SK (2021). MobiHisNet: A lightweight CNN in mobile edge computing for histopathological image classification. IEEE Internet of Things Journal.

[70] Liu Y, Pu H, Sun DW (2021). Efficient extraction of deep image features using convolutional neural network (CNN) for applications in detecting and analysing complex food matrices. Trends in Food Science Technology.

[71] Bhatt D, Patel C, Talsania H, Patel J, Vaghela R (2021). CNN variants for computer vision: History, architecture, application, challenges and future scope. Electronics.

[72] He K, Ji L, Wu CWD, Tso KFG (2021). Using SARIMA-CNN-LSTM approach to forecast daily tourism demand. Journal of Hospitality and Tourism Management.

[73] Sharma R, Sungheetha A (2021). An efficient dimension reduction based fusion of CNN and SVM model for detection of abnormal incident in video surveillance. Journal of Soft Computing Paradigm.

[74] Rodriguez-Ruiz A, Teuwen J, Chung K, Karssemeijer N, Chevalier M (2018). Medical imaging 2018: Computer-aided diagnosis, vol. 10575 105752J.

[75] Wang J, Ding H, Bidgoli FA, Zhou B, Iribarren C (2017). Detecting cardiovascular disease from mammograms with deep learning. IEEE Transactions on Medical Imaging.

[76] Debelee TG, Amirian M, Ibenthal A, Palm G, Schwenker F (2017). Classification of mammograms using convolutional neural network based feature extraction.

[77] Kooi T, van Ginneken B, Karssemeijer N, den Heeten A (2017). Discriminating solitary cysts from soft tissue lesions in mammography using a pretrained deep convolutional neural network. Medical Physics.

[78] Hu Z, Tang J, Wang Z, Zhang K, Zhang L (2018). Deep learning for image-based cancer detection and diagnosis—A survey. Pattern Recognition.

[79] Chittineni S, Edara SS (2022). Machine learning and autonomous systems.

[80] Tripathi RP, Khatri SK, Baxodirovna DVG (2022). A transfer learning approach to implementation ofpretrained CNN models for breast cancer diagnosis. Journal of Positive School Psychology.

[81] Kolchev A, Pasynkov D, Egoshin I, Kliouchkin I, Pasynkova O (2022). YOLOv4-based CNN model versus nested contours algorithm in the suspicious lesion detection on the mammography image: A direct comparison in the real clinical settings. Journal of Imaging.

[82] Liu S, Liu G, Zhou H (2019). A robust parallel object tracking method for illumination variations. Mobile Networks and Applications.

[83] Devika R, Rajasekaran S, Gayathri RL, Priyal J, Kanneganti SR (2022). Automatic breast cancer lesion detection and classification in mammograms using faster R-CNN deep learning network. Issues and Developments in Medicine and Medical Research.

[84] Mahmoud H, Alharbi A, Khafga D (2021). Breast cancer classification using deep convolution neural network with transfer learning. Intelligent Automation Soft Computing.

[85] Liu S, Liu X, Wang S, Muhammad K (2021). Fuzzy-aided solution for out-of-view challenge in visual tracking under IoT-assisted complex environment. Neural Computing and Applications.

[86] Yin W, Kann K, Yu M, Schütze H (2017). Comparative study of CNN and RNN for natural language processing. arXiv preprint.

[87] Hershey S, Chaudhuri S, Ellis DP, Gemmeke JF, Jansen A (2017). CNN architectures for large-scale audio classification.

[88] Wang J, Zhu H, Wang SH, Zhang YD (2021). Are view of deep learning on medical image analysis. Mobile Networks and Applications.

[89] Ge R, Chen G, Saruta K, Terata Y (2021). MDDCNN: Diagnosis of lymph node metastases in breast cancer based on dual-CNN fusion and segmental convolution. Information.

[90] Ranjbarzadeh R, Tataei Sarshar N, Jafarzadeh Ghoushchi S, Saleh Esfahani M, Parhizkar M (2022). MRFE-CNN: Multi-route feature extraction model for breast tumor segmentation in mammograms using a convolutional neural network. Annals of Operations Research.

[91] Sun Y, Xue B, Zhang M, Yen GG, Lv J (2020). Automatically designing CNN architectures using the genetic algorithm for image classification. IEEE Transactions on Cybernetics.

[92] Wei Y, Xia W, Huang J, Ni B, Dong J (2014). CNN: Single-label to multi-label. arXiv preprint.

[93] Ji Y, Zhang H, Zhang Z, Liu M (2021). CNN-based encoder-decoder networks for salient object detection: A comprehensive review and recent advances. Information Sciences.

[94] Chang CC, Lin CJ (2011). LIBSVM: A library for support vector machines. ACM Transactions on Intelligent Systems and Technology.

[95] Gallicchio C, Scardapane S (2020). Deep randomized neural networks. Recent Trends in Learning from Data.

[96] Liu RW, Yuan W, Chen X, Lu Y (2021). An enhanced CNN-enabled learning method for promoting ship detection in maritime surveillance system. Ocean Engineering.

[97] Pradeep S, Nirmaladevi P (2021). A review on speckle noise reduction techniques in ultrasound medical images based on spatial domain, transform domain and CNN methods. IOP Conference Series: Materials Science and Engineering.

[98] Jin N, Wu J, Ma X, Yan K, Mo Y (2020). Multi-task learning model based on multi-scale CNN and LSTM for sentiment classification. IEEE Access.

[99] Yan R, Liao J, Yang J, Sun W, Nong M (2021). Multi-hour and multi-site air quality index forecasting in Beijing using CNN, LSTM, CNN-LSTM, and spatiotemporal clustering. Expert Systems with Applications.

[100] Xiang L, Wang P, Yang X, Hu A, Su H (2021). Fault detection of wind turbine based on SCADA data analysis using CNN and LSTM with attention mechanism. Measurement.

[101] Chen Y, Wang Y, Dong Z, Su J, Han Z (2021). 2-D regional short-term wind speed forecast based on CNN-LSTM deep learning model. Energy Conversion and Management.

[102] Zhang M, Li W, Tao R, Li H, Du Q (2021). Information fusion for classification of hyperspectral and LiDAR data using IP-CNN. IEEE Transactions on Geoscience and Remote Sensing.

[103] Simonyan K, Zisserman A (2014). Very deep convolutional networks for large-scale image recognition. arXiv preprint.

[104] Albawi S, Mohammed TA, Al-Zawi S (2017). Understanding of a convolutional neural network.

[105] Gkioxari G, Malik J, Johnson J (2019). Mesh R-CNN.

[106] Zhang K, Zuo W, Zhang L (2018). FFDNet: Toward a fast and flexible solution for CNN-based image denoising. IEEE Transactions on Image Processing.

[107] Chen K, Wang J, Chen LC, Gao H, Xu W (2015). ABC-CNN: An attention based convolutional neural network for visual question answering. arXiv preprint.

[108] Basiri ME, Nemati S, Abdar M, Cambria E, Acharya UR (2021). ABCDM: An attention-based bidirectional CNN-RNN deep model for sentiment analysis. Future Generation Computer Systems.

[109] Dua N, Singh SN, Semwal VB (2021). Multi-input CNN-GRU based human activity recognition using wearable sensors. Computing.

[110] Zhu Z, Lu S, Wang SH, Górriz JM, Zhang YD (2021). BCNet: A novel network for blood cell classification. Frontiers in Cell and Developmental Biology.

[111] Wu J (2017). Introduction to convolutional neural networks. National Key Lab for Novel Software Technology Nanjing University China.

[112] Chauhan R, Ghanshala KK, Joshi R (2018). Convolutional neural network (CNN) for image detection and recognition.

[113] Hossain MA, Sajib MSA (2019). Classification of image using convolutional neural network (CNN). Global Journal of Computer Science and Technology.

[114] Kido S, Hirano Y, Hashimoto N (2018). Detection and classification of lung abnormalities by use of convolutional neural network (CNN) and regions with CNN features (R-CNN).

[115] Zhang YD, Satapathy SC, Guttery DS, Górriz JM, Wang SH (2021). Improved breast cancer classification through combining graph convolutional network and convolutional neural network. Information Processing Management.

[116] Zhang Q, Wu YN, Zhu SC (2018). Interpretable convolutional neural networks.

[117] Hashemi M (2019). Enlarging smaller images before inputting into convolutional neural network: Zeropadding vs. interpolation. Journal of Big Data.

[118] Fang W, Zhang F, Sheng VS, Ding Y (2018). A method for improving CNN-based image recognition using DCGAN. Computers, Materials Continua.

[119] Zhang M, Li W, Du Q (2018). Diverse region-based CNN for hyperspectral image classification. IEEE Transactions on Image Processing.

[120] Chen D, Bolton J, Manning CD (2016). A thorough examination of the CNN/daily mail reading comprehension task. arXiv preprint.

[121] Hussain M, Bird JJ, Faria DR (2018). A study on CNN transfer learning for image classification.

[122] Akhtar N, Ragavendran U (2020). Interpretation of intelligence in CNN-pooling processes: A methodological survey. Neural Computing and Applications.

[123] Tolias G, Sicre R, Jégou H (2015). Particular object retrieval with integral max-pooling of CNN activations. arXiv preprint.

[124] Gong Y, Wang L, Guo R, Lazebnik S (2014). Multi-scale orderless pooling of deep convolutional activation features.

[125] Vaccaro F, Bertini M, Uricchio T, DelBimbo A (2020). Image retrieval using multi-scale CNN features pooling.

[126] Zhang R, Zhu F, Liu J, Liu G (2019). Depth-wise separable convolutions and multi-level pooling for an efficient spatial CNN-based steganalysis. IEEE Transactions on Information Forensics and Security.

[127] Xiao Y, Wang X, Zhang P, Meng F, Shao F (2020). Object detection based on faster R-CNN algorithm with skip pooling and fusion of contextual information. Sensors.

[128] Giusti A, Cireşan DC, Masci J, Gambardella LM, Schmidhuber J (2013). Fast image scanning with deep max-pooling convolutional neural networks.

[129] Wang S, Jiang Y, Hou X, Cheng H, Du S (2017). Cerebral micro-bleed detection based on the convolution neural network with rank based average pooling. IEEE Access.

[130] Wang S, Sun J, Mehmood I, Pan C, Chen Y (2020). Cerebral micro-bleeding identification basedon a nine-layer convolutional neural network with stochastic pooling. Concurrency and Computation: Practice and Experience.

[131] Hang ST, Aono M (2017). Bi-linearly weighted fractional max pooling. Multimedia Tools and Applications.

[132] Wang SH, Lv YD, Sui Y, Liu S, Wang SJ (2018). Alcoholism detection by data augmentation and convolutional neural network with stochastic pooling. Journal of Medical Systems.

[133] Han J, Moraga C (1995). The influence of the sigmoid function parameters on the speed of backpropa-gation learning.

[134] Fan E (2000). Extended tanh-function method and its applications to nonlinear equations. Physics Letters A.

[135] Agarap AF (2018). Deep learning using rectified linear units (ReLU). arXiv preprint.

[136] Dubey AK, Jain V (2019). Applications of computing, automation and wireless systems in electrical engineering.

[137] Crnjanski J, Krstic’ M, Totovic’ A, Pleros N, Gvozdic’ D (2021). Adaptive sigmoid-like and PReLU activation functions for all-optical perceptron. Optics Letters.

[138] Santurkar S, Tsipras D, Ilyas A, Madry A (2018). How does batch normalization help optimization?. Advances in Neural Information Processing Systems.

[139] Wu H, Gu X (2015). Towards dropout training for convolutional neural networks. Neural Networks.

[140] Behar N, Shrivastava M (2022). ResNet50-based effective model for breast cancer classification using histopathology images. Computer Modeling in Engineering Sciences.

[141] Alkhaleefah M, Wu CC (2018). A hybrid CNN and RBF-based SVM approach for breast cancer classification in mammograms.

[142] Liu K, Kang G, Zhang N, Hou B (2018). Breast cancer classification based on fully-connected layer first convolutional neural networks. IEEE Access.

[143] Gour M, Jain S, Sunil Kumar T (2020). Residual learning based CNN for breast cancer histopathological image classification. International Journal of Imaging Systems and Technology.

[144] Wang Y, Sun L, Ma K, Fang J (2018). Breast cancer microscope image classification based on CNN with image deformation.

[145] Yao H, Zhang X, Zhou X, Liu S (2019). Parallel structure deep neural network using CNN and RNN with an attention mechanism for breast cancer histology image classification. Cancers.

[146] Agnes SA, Anitha J, Pandian S, Peter JD (2020). Classification of mammogram images using multiscale all convolutional neural network (MA-CNN). Journal of Medical Systems.

[147] Wang Y, Choi EJ, Choi Y, Zhang H, Jin GY (2020). Breast cancer classification in automated breast ultrasound using multiview convolutional neural network with transfer learning. Ultrasound in Medicine Biology.

[148] Saikia AR, Bora K, Mahanta LB, Das AK (2019). Comparative assessment of CNN architectures for classification of breast FNAC images. Tissue and Cell.

[149] Mewada HK, Patel AV, Hassaballah M, Alkinani MH, Mahant K (2020). Spectral-spatial features integrated convolution neural network for breast cancer classification. Sensors.

[150] Zhou Y, Xu J, Liu Q, Li C, Liu Z (2018). A radiomics approach with CNN for shear-wave elastography breast tumor classification. IEEE Transactions on Biomedical Engineering.

[151] Lotter W, Sorensen G, Cox D (2017). Deep learning in medical image analysis and multimodal learning for clinical decision support.

[152] Vidyarthi A, Shad J, Sharma S, Agarwal P (2019). Classification of breast microscopic imaging using hybrid CLAHE-CNN deep architecture.

[153] Hijab A, Rushdi MA, Gomaa MM, Eldeib A (2019). Breast cancer classification in ultrasound images using transfer learning.

[154] Kumar K, Rao ACS (2018). Breast cancer classification of image using convolutional neural network.

[155] Kousalya K, Saranya T (2021). Improved the detection and classification of breast cancer using hyper parameter tuning. Materials Today: Proceedings.

[156] Mikhailov N, Shakeel M, Urmanov A, Lee MH, Demirci MF (2021). Optimization of CNN model for breast cancer classification.

[157] Karthik R, Menaka R, Kathiresan G, Anirudh M, Nagharjun M (2021). Gaussian dropout based stacked ensemble CNN for classification of breast tumor in ultrasound images. IRBM.

[158] Nawaz M, Sewissy AA, Soliman THA (2018). Multi-class breast cancer classification using deep learning convolutional neural network. International Journal of Advanced Computer Science and Applications.

[159] Deniz E, Sengür A, Kadiroglu Z, Guo Y, Bajaj V (2018). Transfer learning based histopatho-logic image classification for breast cancer detection. Health Information Science and Systems.

[160] Yeh JY, Chan S (2018). CNN-based CAD for breast cancer classification in digital breast tomosynthesis.

[161] Gonçalves CB, Souza JR, Fernandes H (2021). Classification of static infrared images using pretrained CNN for breast cancer detection.

[162] Bayramoglu N, Kannala J, Heikkilä J (2016). Deep learning for magnification independent breast cancer histopathology image classification.

[163] Alqahtani Y, Mandawkar U, Sharma A, Hasan MNS, Kulkarni MH (2022). Breast cancer pathological image classification based on the multiscale CNN squeeze model. Computational Intelligence and Neuroscience.

[164] Sharma R, Sharma JB, Maheshwari R, Agarwal P (2022). Thermogram adaptive efficient model for breast cancer detection using fractional derivative mask and hybrid feature set in the IoT environment. Computer Modeling in Engineering Sciences.

[165] Sohail A, Khan A, Wahab N, Zameer A, Khan S (2021). A multi-phase deep CNN based mitosis detection framework for breast cancer histopathological images. Scientific Reports.

[166] Mahmood T, Arsalan M, Owais M, Lee MB, Park KR (2020). Artificial intelligence-based mitosis detection in breast cancer histopathology images using faster R-CNN and deep CNNs. Journal of Clinical Medicine.

[167] Wang Z, Li M, Wang H, Jiang H, Yao Y (2019). Breast cancer detection using extreme learning machine based on feature fusion with CNN deep features. IEEE Access.

[168] Chiao JY, Chen KY, Liao KYK, Hsieh PH, Zhang G (2019). Detection and classification the breast tumors using mask R-CNN on sonograms. Medicine.

[169] Das K, Conjeti S, Chatterjee J, Sheet D (2020). Detection of breast cancer from whole slide histopathological images using deep multiple instance CNN. IEEE Access.

[170] Zainudin Z, Shamsuddin SM, Hasan S (2019). Deep layer CNN architecture for breast cancer histopathology image detection.

[171] Wu B, Kausar T, Xiao Q, Wang M, Wang W (2017). FF-CNN: An efficient deep neural network for mitosis detection in breast cancer histological images.

[172] Gonçalves CB, de Souza JR, Fernandes H (2022). CNN architecture optimization using bio-inspired algorithms for breast cancer detection in infrared images. Computers in Biology and Medicine.

[173] Guan S, Loew M (2019). Medical imaging 2019, imaging informatics for healthcare, research, and applications.

[174] Hadush S, Girmay Y, Sinamo A, Hagos G (2020). Breast cancer detection using convolutional neural networks. arXiv preprint.

[175] Huang J, Mei L, Long M, Liu Y, Sun W (2022). BM-Net: CNN-based MobileNet-V3 and bilinear structure for breast cancer detection in whole slide images. Bioengineering.

[176] Mahbub TN, Yousuf MA, Uddin MN (2022). A modified CNN and fuzzy AHP based breast cancer stage detection system.

[177] Prajoth SenthilKumar A, Narendra M, Jani Anbarasi L, Raj BE (2021). Breast cancer analysis and detection in histopathological images using CNN approach.

[178] Charan S, Khan MJ, Khurshid K (2018). Breast cancer detection in mammograms using convolutional neural network.

[179] Alanazi SA, Kamruzzaman M, Islam Sarker MN, Alruwaili M, Alhwaiti Y (2021). Boosting breast cancer detection using convolutional neural network. Journal of Healthcare Engineering.

[180] Gonçalves CB, Souza JR, Fernandes H (2022). CNN optimization using surrogate evolutionary algorithm for breast cancer detection using infrared images.

[181] Guan S, Loew M (2019). Breast cancer detection using synthetic mammograms from generative adversarial networks in convolutional neural networks. Journal of Medical Imaging.

[182] Sun L, Sun H, Wang J, Wu S, Zhao Y (2021). Breast mass detection in mammography based on image template matching and CNN. Sensors.

[183] Chauhan A, Kharpate H, Narekar Y, Gulhane S, Virulkar T (2021). Breast cancer detection and prediction using machine learning.

[184] Gupta V, Vasudev M, Doegar A, Sambyal N (2021). Breast cancer detection from histopathology images using modified residual neural networks. Biocybernetics and Biomedical Engineering.

[185] Chouhan N, Khan A, Shah JZ, Hussnain M, Khan MW (2021). Deep convolutional neural network and emotional learning based breast cancer detection using digital mammography. Computers in Biology and Medicine.

[186] Chen X, Men K, Chen B, Tang Y, Zhang T (2020). CNN-Based quality assurance for automatic segmentation of breast cancer in radiotherapy. Frontiers in Oncology.

[187] El Adoui M, Mahmoudi SA, Larhmam MA, Benjelloun M (2019). MRI breast tumor segmentation using different encoder and decoder CNN architectures. Computers.

[188] Kakileti ST, Manjunath G, Madhu HJ (2019). Cascaded CNN for view independent breast segmentation in thermal images.

[189] Kumar MN, Jatti A, Narayanappa C (2019). Probable region identification and segmentation in breast cancer using the DL-CNN.

[190] Atrey K, Singh BK, Roy A, Bodhey NK (2021). Real-time automated segmentation of breast lesions using CNN-based deep learning paradigm: Investigation on mammogram and ultrasound. International Journal of Imaging Systems and Technology.

[191] Irfan R, Almazroi AA, Rauf HT, Damasevicius R, Nasr EA (2021). Dilated semantic segmentation for breast ultrasonic lesion detection using parallel feature fusion. Diagnostics.

[192] Su H, Liu F, Xie Y, Xing F, Meyyappan S (2015). Region segmentation in histopathological breast cancer images using deep convolutional neural network.

[193] He S, Ruan J, Long Y, Wang J, Wu C (2018). Combining deep learning with traditional features for classification and segmentation of pathological images of breast cancer.

[194] Soltani H, Amroune M, Bendib I, Haouam MY (2021). Breast cancer lesion detection and segmentation based on mask R-CNN.

[195] Min H, Wilson D, Huang Y, Liu S, Crozier S (2020). Fully automatic computer-aided mass detection and segmentation via pseudo-color mammograms and mask R-CNN.

[196] Arora R, Raman B (2021). A deep neural CNN model with CRF for breast mass segmentation in mammograms.

[197] Spuhler KD, Ding J, Liu C, Sun J, Serrano-Sosa M (2019). Task-based assessment of a convolutional neural network for segmenting breast lesions for radiomic analysis. Magnetic Resonance in Medicine.

[198] Atrey K, Singh BK, Roy A, Bodhey NK (2022). Real-time automated segmentation of breastlesions using CNN-based deep learning paradigm: Investigation on mammogram and ultrasound. International Journal of Imaging Systems and Technology.

[199] Sumathi R, Vasudevan V (2022). Intelligent systems and sustainable computing.

[200] Xu Y, Wang Y, Yuan J, Cheng Q, Wang X (2019). Medical breast ultrasound image segmentation by machine learning. Ultrasonics.

[201] Guo YY, Huang YH, Wang Y, Huang J, Lai QQ (2022). Breast MRI tumor automatic segmentation and triple-negative breast cancer discrimination algorithm based on deep learning. Computational and Mathematical Methods in Medicine.

[202] Cui Z, Yang J, Qiao Y (2016). Brain MRI segmentation with patch-based CNN approach.

